# Urban Weeds: Supporters of Pollinator Biodiversity in Urban Landscapes

**DOI:** 10.3390/plants15182765

**Published:** 2026-09-09

**Authors:** Stefano Benvenuti

**Affiliations:** Department of Agri-Food Sciences and Technologies, University of Bologna, Viale Giuseppe Fanin, 46, 40127 Bologna, Italy; stefano.benvenuti8@unibo.it

**Keywords:** urban biodiversity, ecosystem services, plant–pollinator interactions, wild flora

## Abstract

Urbanization is widely recognized as a major driver of biodiversity loss; however, cities can also provide important refuges for pollinators when diverse and continuous floral resources are available. This study assessed the diversity of spontaneous insect-pollinated flora and its associated pollinator communities across five major urban habitat types (grasslands, road verges, artificial surfaces, wooded areas, and wetlands) in the Mediterranean cities of Pisa, Livorno, and Lucca (central Italy). During a three-year field survey, spontaneous flowering plant species were systematically recorded, and pollinator visitation rates were quantified using standardized sampling protocols. Approximately 150 spontaneous insect-pollinated plant species were identified, providing a continuous supply of nectar and pollen throughout the flowering season. Pollinator assemblages included honey bees (*Apis mellifera*), solitary bees, bumblebees, Diptera, and Lepidoptera, with bees representing the dominant group across all habitats. Pollinator visitation rates differed significantly among habitat types, reaching their highest values in grasslands, road verges, and wetlands, whereas wooded habitats and artificial surfaces supported significantly lower pollinator activity. Numerous plant species belonging to diverse botanical families attracted a broad range of pollinator taxa, revealing complex plant–pollinator interactions and demonstrating the ecological value of spontaneous urban vegetation for sustaining pollinator diversity. In summary, the present study shows that spontaneous flora constitutes a fundamental component of urban green infrastructure, supporting pollination services and enhancing ecological connectivity within cities. Biodiversity-friendly management strategies that conserve and promote spontaneous flowering vegetation should therefore be considered a key element of sustainable urban planning and the ecological resilience of Mediterranean urban landscapes.

## 1. Introduction

The concept of a weed is entirely anthropocentric, as no plant species is intrinsically undesirable. Rather, whether a species is regarded as a weed depends exclusively on human objectives and on the way a particular environment is managed. In urban ecosystems, spontaneous vegetation has traditionally been perceived as undesirable because it may reduce the aesthetic appearance of built environments, interfere with the functionality and accessibility of pavements, artificial surfaces and recreational green spaces, or, in the case of some wind-pollinated species, represent a public health concern owing to the production of allergenic airborne pollen [1]. Consequently, urban vegetation management has historically been based on the implicit assumption that the complete removal of weed vegetation is synonymous with improved urban quality.

During the last two decades, this traditional perception has progressively changed as increasing attention has been devoted to urban biodiversity, ecosystem services and nature-based solutions [2]. Rather than considering all wild vegetation equally undesirable, current ecological approaches recognize that urban plant species differ greatly in the balance between their potential negative impacts and the ecological benefits they provide. Consequently, the objective of urban vegetation management is gradually shifting from indiscriminate weed eradication towards the selective conservation of species that enhance ecosystem functioning while controlling those that genuinely compromise human health, safety or infrastructure.

Among the ecosystem services provided by wild urban vegetation, those associated with insect-pollinated species are particularly important yet frequently overlooked. Spontaneous flowering plants contribute to supporting biodiversity by supplying nectar and pollen resources for pollinating insects, increasing ecological connectivity among urban habitats and sustaining trophic interactions across multiple taxonomic groups [3]. They also contribute to regulating ecological processes and improving the resilience of urban ecosystems [4]. In addition, wild flowering vegetation provides important cultural ecosystem services by enriching the visual quality of cities through the seasonal succession of colours, floral forms and scents. Unlike ornamental species intentionally planted in urban landscapes, these benefits are delivered spontaneously and continuously, often with little or no economic investment for establishment and maintenance.

The remarkable attractiveness of insect-pollinated flowers is itself the result of a long evolutionary history. Through millions of years of co-evolution between flowering plants and their pollinators, natural selection has favoured increasingly conspicuous flowers characterized by bright colours, complex corolla architectures, floral scents and rewards such as nectar and pollen that maximize pollinator attraction and reproductive success [5]. Floral beauty, therefore, is not merely an aesthetic quality appreciated by humans but primarily an adaptive evolutionary strategy shaped by pollinator-mediated selection. Interestingly, the same floral traits that attract bees, bumblebees, butterflies, hoverflies and other pollinating insects are also those that generate many of the aesthetic perceptions that humans associate with attractive urban landscapes.

By sustaining pollinator communities, insect-pollinated urban weeds also support complex urban food webs. Flowering plants constitute the primary producers, pollinating insects represent an essential trophic component, and numerous insectivorous birds and other vertebrates ultimately depend upon these interactions [6]. Consequently, even relatively small patches of wild flowering vegetation may contribute disproportionately to maintaining urban biodiversity and ecological resilience.

Beyond their ecological role, wild flowering plants also provide important cultural ecosystem services that directly affect human well-being. Growing evidence indicates that regular exposure to biodiverse urban environments reduces psychological stress, improves mental health and strengthens people’s connection with nature [7]. Small populations of flowering weeds occurring along road verges, grassy areas and other urban habitats introduce seasonal dynamics into otherwise static artificial landscapes, allowing citizens to perceive the progression of the seasons through continuously changing colours accompanied by the activity of bees, butterflies and other pollinating insects. These subtle but recurrent interactions with urban nature contribute to making cities more liveable, attractive and psychologically restorative.

Within this broader perspective, the traditional objective of maintaining completely weed-free urban environments is progressively being replaced by a more evidence-based approach centred on distinguishing species according to their actual ecological impacts. While the presence of highly allergenic wind-pollinated species such as *Parietaria officinalis* and *Ambrosia artemisiifolia* legitimately requires control because of their abundant airborne pollen [8], most insect-pollinated species produce comparatively large and heavy pollen grains adapted for animal transport rather than wind dispersal. Consequently, they generally present a much lower risk of causing airborne allergies while simultaneously providing essential floral resources that sustain pollinator populations throughout the growing season [9].

The ecological importance of wild flowering vegetation has become even more evident in the context of the worldwide decline of pollinating insects. Habitat loss, agricultural intensification, pesticide use and landscape homogenization have drastically reduced the abundance and diversity of floral resources available in many agroecosystems [10]. Intensive agricultural systems frequently lack semi-natural habitats such as hedgerows, uncultivated field margins, species-rich grasslands and woodland patches that are essential for maintaining pollinator populations. Under these conditions, urban ecosystems may paradoxically function as important refuges for pollinators. Although cities are characterized by high levels of anthropogenic disturbance, they are generally subjected to lower agricultural pesticide pressure and often contain a heterogeneous mosaic of habitats capable of providing continuous floral resources over extended periods of the year [11].

The growing interest in urban beekeeping further supports the recognition of cities as valuable habitats for pollinator conservation. During recent years, apiaries have been established in numerous metropolitan areas throughout Europe, North America, Asia, Africa and Australia, not only for the production of urban honey but also as indicators of ecological quality and as effective tools for biodiversity conservation and environmental education [12]. Plant–pollinator mutualisms represent one of the most visible expressions of urban biodiversity and provide an effective means of promoting ecological awareness and reconnecting citizens with nature.

Although the contribution of ornamental herbaceous plants, shrubs and trees to urban pollinator conservation has been widely investigated [13], wild herbaceous flora has received considerably less attention despite comprising a remarkably diverse assemblage of species differing in flowering phenology, floral morphology, nectar and pollen production, flowering duration and attractiveness to different pollinator guilds. Since urban weed management is inevitably species-selective, identifying those wild species that provide the greatest ecological benefits is essential for developing management strategies capable of reconciling biodiversity conservation, ecosystem service provision and the functional requirements of urban environments.

Furthermore, not all insect-pollinated weeds contribute equally to pollinator conservation. Some species receive exceptionally high visitation rates and may function as keystone floral resources during periods of limited flower availability, whereas others play only a marginal ecological role [14]. Quantifying pollinator visitation therefore provides a robust ecological criterion for identifying the wild species that deserve priority conservation within different urban habitats and for supporting evidence-based urban vegetation management.

The objective of the present study was to identify the most widespread wild insect-pollinated plant species occurring across the principal urban habitats of Mediterranean cities and to evaluate their importance as floral resources through a three-year field survey. Pollinator visitation rates were quantified under standardized field conditions to identify the species that contribute most to sustaining urban pollinator communities. The results provide a scientific basis for distinguishing beneficial from harmful urban weeds and for promoting selective management strategies capable of simultaneously enhancing biodiversity conservation, ecosystem service delivery and the ecological quality of urban environments.

Accordingly, the study focused on identifying the plant species and urban habitats that make the greatest contribution to sustaining pollinator communities, thereby providing an ecological basis for evidence-based urban vegetation management.

Particular attention was devoted to comparing the five principal urban habitat types in terms of spontaneous flowering vegetation, flowering phenology, and pollinator visitation patterns in order to evaluate their complementary contribution to urban pollinator conservation.

## 2. Materials and Methods

Following more than ten years of preliminary observations on the wild flora of urban ecosystems [15], a three-year field survey (2023–2025) was specifically designed to investigate insect-pollinated urban weeds and their interactions with flower-visiting insects. The study was conducted in the urban ecosystems of Pisa (43.7167° N, 10.4000° E), Livorno (43.5500° N, 10.3167° E) and Lucca (43.8429° N, 10.5027° E), Tuscany, central Italy, all characterized by a Mediterranean climate. The survey encompassed the principal urban habitat types, including artificial surfaces, road verges, grassy areas, shaded sites beneath urban tree canopies, and wetlands. Representative sites for each urban habitat type were selected on the basis of their occurrence, accessibility, and the presence of well-established spontaneous flowering vegetation, in order to encompass the typical characteristics of each habitat across the study area. Within each habitat, naturally occurring flowering patches of wild species showing clear adaptations to insect pollination, such as conspicuous corollas and showy inflorescences, were selected throughout the flowering season. Flowering patches were selected only when they were dominated by a single species to avoid interference from neighbouring floral resources. For each flowering plant species, three spatially independent flowering patches were selected as replicate sampling units. Pollinator visitation was assessed separately in each of the three patches using the standardized quadrat method described below.

For each flowering species, the following information was recorded: (i) botanical identification, (ii) pollinator visitation rate, and (iii) the predominant taxonomic groups of flower visitors. Pollinating insects were classified into five main categories: honey bees (*Apis mellifera* L.), solitary bees, bumblebees (*Bombus* spp.), Diptera (hoverflies, tachinid flies and bee flies), and Lepidoptera.

Plant families are reported according to the Cronquist classification system to maintain consistency with the floristic framework adopted throughout this study.

Field observations were performed exclusively under favourable weather conditions (sunny days without appreciable wind) between 10:00 and 18:00 h, corresponding to the period of maximum pollinator activity. For each plant species, pollinator visitation was quantified by placing a 30 × 30 cm quadrat over the flowering patch and recording all insect visits to flowers or inflorescences within the sampled area during a 5 min observation period. Visitation rates were subsequently standardized and expressed as the number of pollinator visits m^−2^ h^−1^. Within each habitat, naturally occurring flowering patches of wild species showing clear adaptations to insect pollination, such as conspicuous corollas and showy inflorescences, were selected. Because the investigated species differed markedly in growth habit, plant architecture and population density, pollinator activity was standardized per unit area rather than per individual plant, allowing meaningful comparisons among species. A pollinator visit was defined as an insect landing on a flower or inflorescence and making contact with the reproductive structures. Surveys were conducted from March to October, according to the flowering phenology of each species. For each plant species, the three independent flowering patches were surveyed during each growing season. The same sampling procedure was repeated over the three study years (2023–2025), resulting in nine patch-level observations per species (three independent patches × three years). The three flowering patches therefore represented the spatial replicates of the sampling design, whereas the three study years represented repeated annual sampling periods. Visitation rates were subsequently standardized and expressed as the number of pollinator visits per square metre per hour (visits m^−2^ h^−1^), allowing direct comparison among plant species and urban habitats. To compare the attractiveness of the wild flora associated with the different urban habitats, the mean pollinator visitation rate of the recorded plant species was calculated and expressed as the number of insect visits m^−2^ h^−1^. In addition, all pollinator observations were pooled across plant species and habitats, and the total number of individuals recorded for each pollinator group was expressed as a percentage of the overall pollinator community. This analysis was used to identify the relative contribution and predominance of the major pollinator taxa within the urban ecosystem. Data were analysed by one-way ANOVA, and treatment means were separated using Tukey’s honestly significant difference (HSD) test at *p* < 0.05. Differences in pollinator attractiveness among the five urban habitat types were assessed using the mean pollinator visitation rates (visits m^−2^ h^−1^) of the recorded wild plant species. Statistical analyses were performed using R version 4.4.0 (R Core Team, Vienna, Austria).

## 3. Results and Discussion

The comparison of the five urban habitat types showed that each supported a characteristic assemblage of spontaneous flowering plants and pollinator visitors. Differences in species composition and flowering phenology resulted in a complementary availability of floral resources throughout the flowering season, highlighting the ecological importance of maintaining a heterogeneous urban habitat mosaic.

### 3.1. Cement-Based Built Environments: Walls, Roofs, and Sidewalks

**Figure 1:**
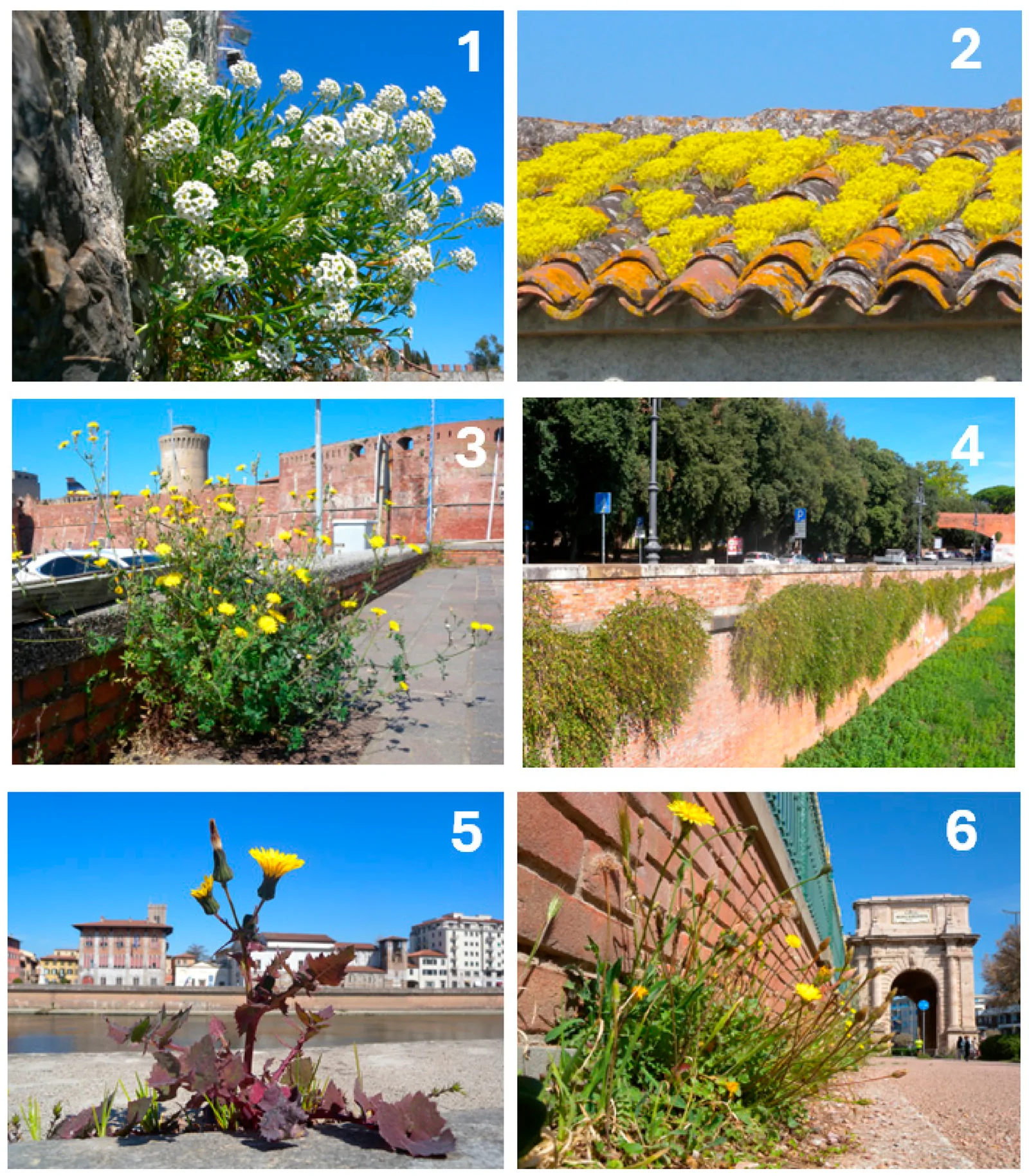
Insect-pollinated plant species ((**1**) *Lobularia maritima*, (**2**) *Sedum rupestre*, (**3**) *Sonchus tenerrimus*, (**4**) *Capparis spinosa*, (**5**) *Sonchus asper*, (**6**) *Hyoseris radiata*) recorded in different cemented habitats (sidewalks, walls, and rooftops) across the three urban ecosystems studied ((**1**,**4**,**5**) Pisa; (**2**) Lucca; (**3**) and (**6**) Livorno).

Despite representing one of the most heavily disturbed urban habitats, built environments (Figure 1) supported a remarkably diverse assemblage of insect-pollinated wild plant species (Table 1). This finding confirms that cities are not ecological deserts but heterogeneous environments capable of supporting a rich diversity of flowering plants and pollinating insects despite intense anthropogenic disturbance [16].

The predominance of Asteraceae, together with the high frequency of therophytes and chamaephytes, reflects the strong environmental filtering imposed by shallow substrates, prolonged summer drought and elevated surface temperatures. These biological traits are characteristic of spontaneous vegetation colonizing artificial Mediterranean habitats, where species adapted to recurrent disturbance and limited water availability are selectively favoured [17].

One of the most important ecological features observed in the present study was the almost continuous succession of flowering from early spring to late autumn. Continuous floral resource availability is considered essential for sustaining pollinator populations because it minimizes seasonal resource shortages and allows different pollinator guilds to complete their life cycles successfully [18]. Consequently, early-flowering species such as *Cerastium glomeratum*, *Fumaria capreolata* and *Veronica cymbalaria*, together with late-flowering species such as *Calamintha nepeta*, *Diplotaxis tenuifolia*, *Oxalis corniculata* and especially *Inula viscosa*, may substantially increase the ecological value of urban habitats by extending the temporal availability of nectar and pollen [19].

The marked differences in pollinator visitation rates among plant species further demonstrate that spontaneous urban flora does not contribute equally to pollinator conservation. Species such as *Inula viscosa*, *Centranthus ruber*, *Crepis sancta*, *Reichardia picroides* and *Sedum rupestre* attracted exceptionally high numbers of flower visitors, suggesting that they function as key floral resources within urban ecosystems. Their conservation could therefore provide disproportionately greater ecological benefits than the preservation of less attractive species [20].

Differences in the dominant pollinator guilds among plant species also indicate a high degree of functional complementarity within urban plant–pollinator networks. Solitary bees, honey bees, bumblebees, hoverflies and Lepidoptera showed distinct preferences for different floral resources, suggesting that variation in flower morphology, phenology and reward characteristics promotes resource partitioning among pollinator groups and contributes to the stability of pollination networks [21].

The present findings demonstrate that spontaneous vegetation colonizing built environments should no longer be regarded simply as undesirable urban weeds. Instead, many insect-pollinated species provide valuable ecosystem services by supporting diverse pollinator communities, maintaining floral resources throughout the growing season and enhancing urban biodiversity. These results support the adoption of selective vegetation management strategies aimed at conserving highly beneficial flowering species while restricting control measures only to those species that genuinely threaten public health, infrastructure or urban safety [22].

### 3.2. Roadside Verges

**Figure 2:**
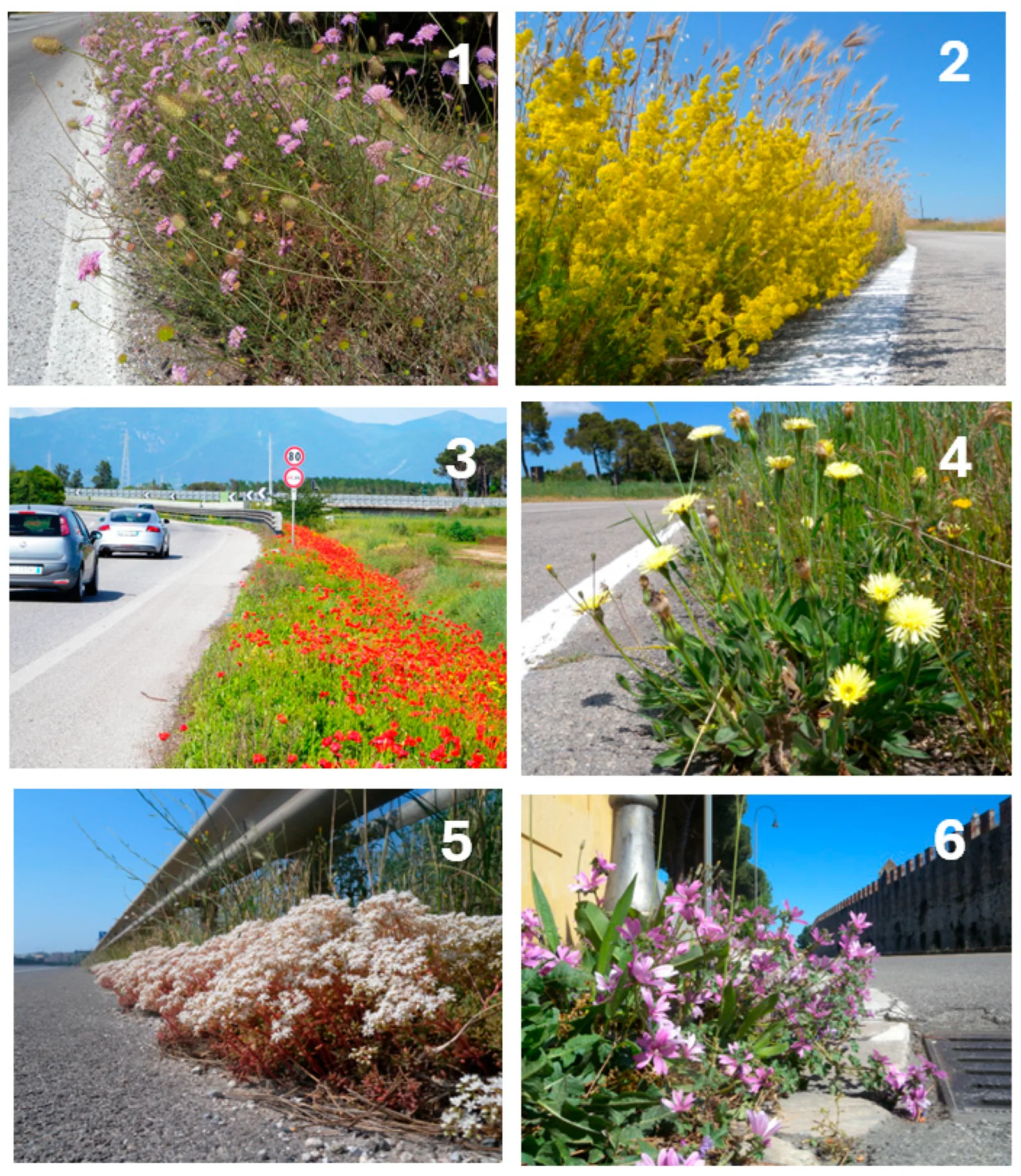
Road verges where insect-pollinated plant species ((**1**) *Scabiosa columbaria*, (**2**) *Galium verum*, (**3**) *Papaver rhoeas*, (**4**) *Urospermum dalechampii*, (**5**) *Sedum album*, (**6**) *Malva sylvestris*) were recorded across the three urban ecosystems studied ((**1**,**2**,**5**) Livorno; (**3**) and (**6**) Pisa; (**4**) Lucca).

Roadside verges supported one of the richest assemblages of insect-pollinated wild plant species (Figure 2) recorded in the present study (Table 2), confirming that these linear habitats can represent important reservoirs of biodiversity within urban landscapes despite being subjected to frequent anthropogenic disturbance [23].

The predominance of hemicryptophytes reflects the intermediate disturbance regime typically associated with roadside management, where periodic mowing prevents woody succession while allowing the persistence of perennial herbaceous communities. Such management practices create structurally diverse habitats capable of supporting a high diversity of flowering species and associated insects [24].

The prolonged flowering period, extending from early spring to early autumn, considerably enhanced the ecological value of roadside verges by ensuring a continuous supply of nectar and pollen resources. Temporal continuity of floral resources is widely recognized as one of the principal factors regulating pollinator abundance and diversity, particularly in fragmented urban landscapes where foraging opportunities may be spatially and temporally limited [25].

The exceptionally high visitation rates recorded for *Cephalaria transsylvanica*, *Scabiosa columbaria*, *Hedysarum coronarium*, *Lotus corniculatus*, *Trifolium repens* and *Cichorium intybus* indicate that these species function as highly attractive floral resources capable of sustaining large numbers of pollinating insects. Their conservation within roadside vegetation could therefore substantially enhance the ecological functionality of these habitats while simultaneously increasing pollination services across the urban landscape [26].

The predominance of solitary bees on many of the most attractive flowering species further emphasizes the ecological importance of roadside verges for wild bee conservation. Unlike managed honey bees, solitary bees generally exhibit more specialized nesting and foraging requirements and are often considered particularly sensitive indicators of habitat quality. The abundance of suitable floral resources along road verges may therefore contribute significantly to maintaining diverse wild bee communities within cities [27].

The occurrence of different dominant pollinator guilds among plant species, including honey bees, bumblebees, hoverflies, bee flies and tachinid flies, further demonstrates the functional diversity of roadside plant–pollinator interactions. Differences in floral morphology, phenology and resource availability promote complementary resource use among pollinator taxa, thereby increasing the stability and resilience of urban pollination networks [28].

Within road verges, pollinator visitation was concentrated on a limited number of plant species, which showed the highest visitation rates recorded in this habitat. When appropriately managed, they can function as valuable ecological corridors, enhancing habitat connectivity, supporting pollinator diversity and contributing substantially to biodiversity conservation within urban ecosystems [29].

### 3.3. Urban Herbaceous Patches and Grasslands

**Figure 3:**
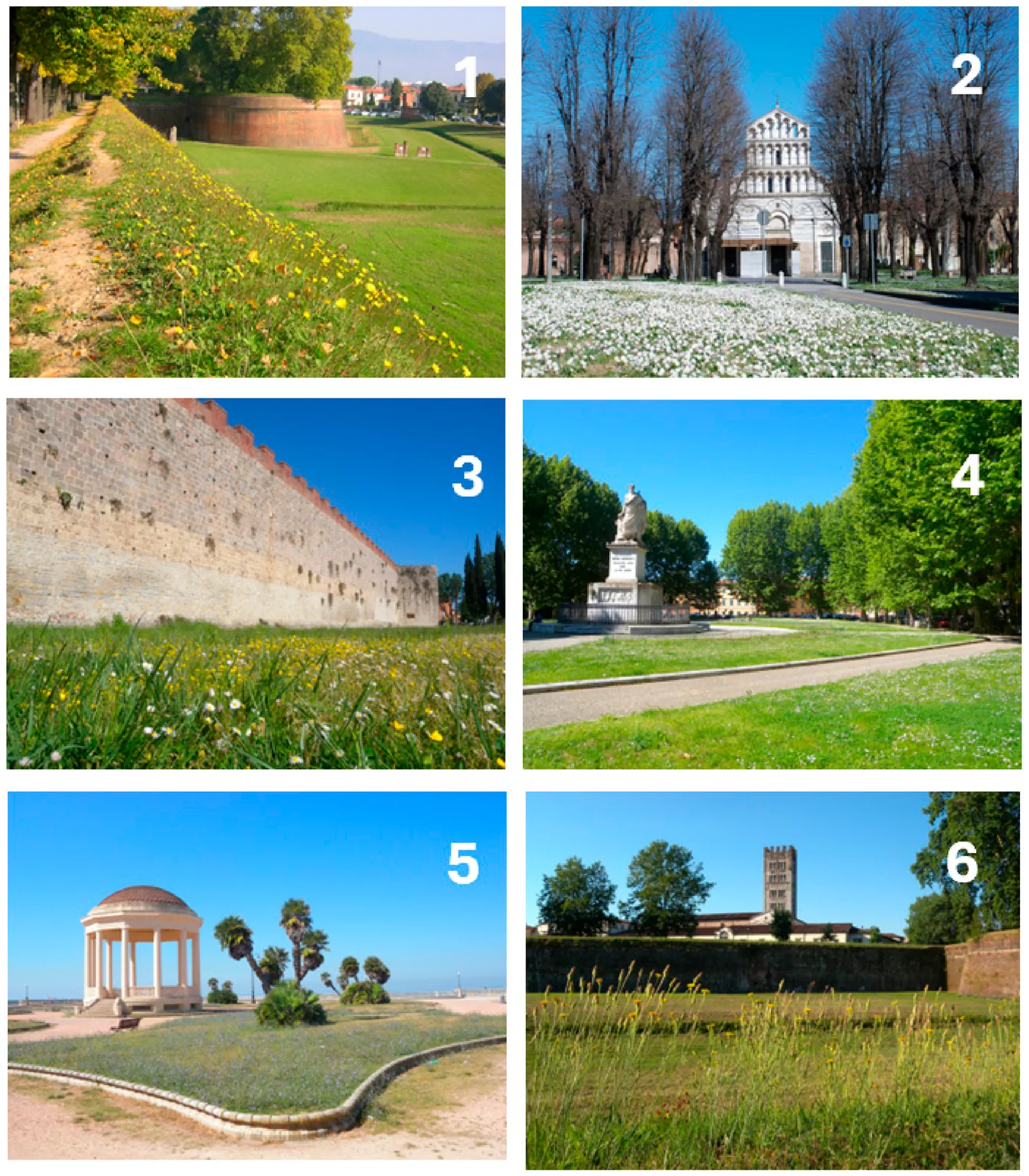
Grassland habitats where insect-pollinated plant species were recorded across the three urban ecosystems studied ((**1**,**6**) Lucca; (**2**–**4**) Pisa; (**5**) Livorno).

Urban herbaceous patches and grasslands supported one of the most diverse assemblages of insect-pollinated wild plant (Figure 3) species recorded during the study (Table 3), confirming the important role of these semi-natural habitats in maintaining urban biodiversity. Their high floristic diversity provides a wide range of floral traits and ecological niches capable of supporting taxonomically diverse pollinator communities [30].

The predominance of hemicryptophytes reflects the relatively stable ecological conditions of perennial grassland communities, whereas the presence of numerous therophytes contributes to increasing seasonal floristic turnover. This combination of life forms promotes structural and temporal heterogeneity, which is widely recognized as a key determinant of biodiversity in semi-natural grasslands [31].

A particularly important feature of these habitats was the continuous succession of flowering species from early spring until late summer. Such prolonged flowering periods ensure a stable supply of nectar and pollen throughout the entire period of pollinator activity, thereby reducing seasonal fluctuations in food availability and increasing the capacity of urban grasslands to sustain abundant and diverse pollinator assemblages [32].

Several species, including *Trifolium pratense*, *Cirsium arvense*, *Crepis vesicaria*, *Ornithogalum umbellatum*, *Echium vulgare* and *Taraxacum officinale*, exhibited exceptionally high visitation rates, indicating that they represent particularly valuable floral resources. Their conservation within urban grasslands could therefore substantially enhance pollination services while supporting the persistence of both generalist and specialist pollinators [33].

The balanced representation of honey bees, solitary bees, bumblebees, hoverflies, bee flies and Lepidoptera further demonstrates the high functional diversity of these habitats. Different pollinator guilds exhibited distinct preferences for particular plant species, suggesting that variation in flowering phenology, floral architecture and resource quality promotes complementary exploitation of floral resources and contributes to the resilience of urban pollination networks [34].

The present results highlight urban herbaceous patches and grasslands as biodiversity hotspots within the urban matrix. Their combination of high plant diversity, prolonged flowering and functionally diverse pollinator communities suggests that these habitats should represent a priority for conservation and ecologically based management. Maintaining low-intensity mowing regimes and preserving spontaneous flowering vegetation could substantially enhance ecosystem services and strengthen pollinator conservation in urban landscapes [35].

### 3.4. Areas Shaded by Urban Forests

**Figure 4:**
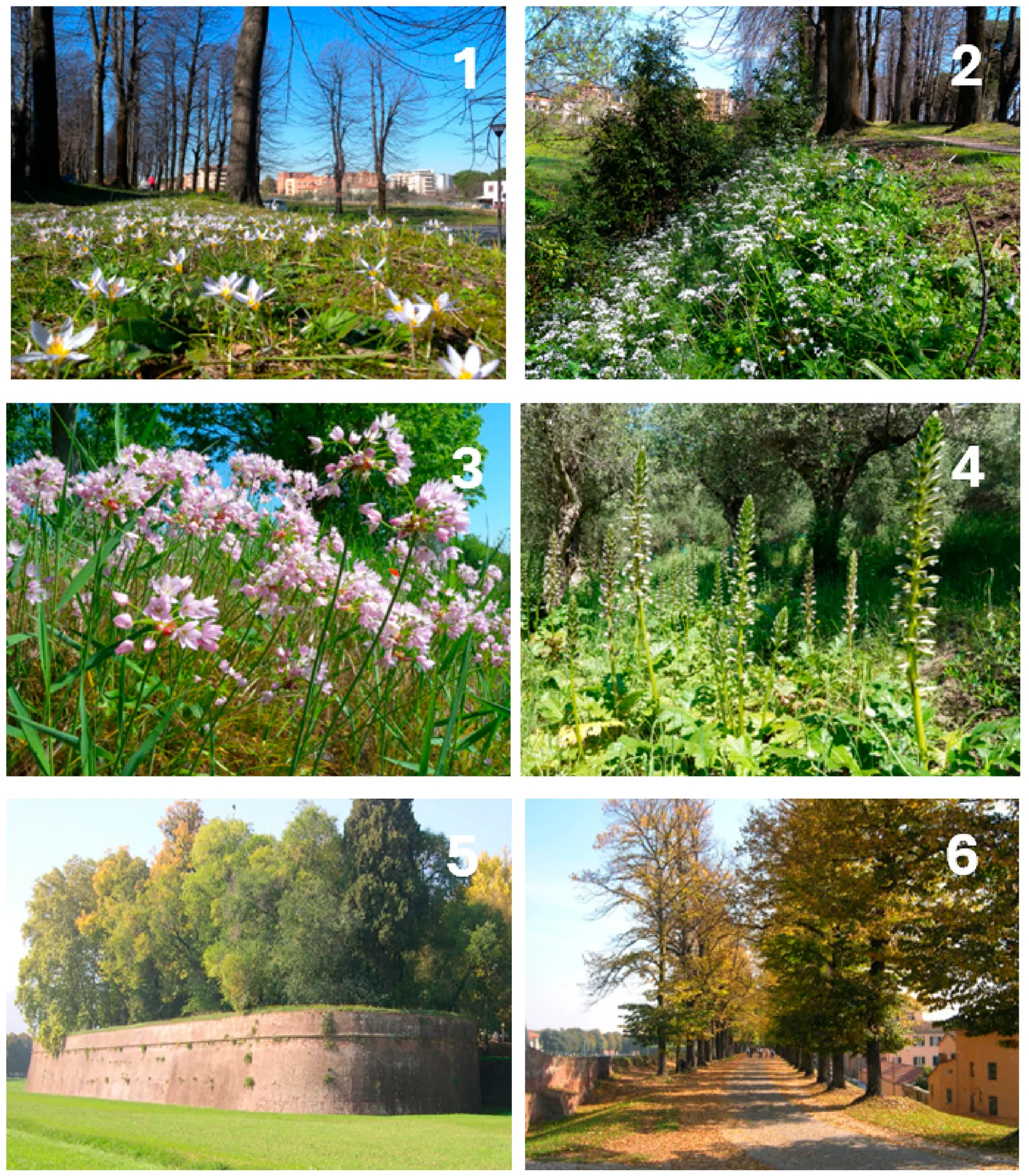
Shaded habitats where insect-pollinated plant species were recorded across the three urban ecosystems studied ((**1**,**2**) Pisa; (**3**,**4**) Livorno; (**5**,**6**) Lucca). In the first four images, the distinctive forms and colours of *Romulea bulbocodium* (**1**), *Allium neapolitanum* (**2**), *Allium roseum* (**3**), and *Acanthus mollis* (**4**) are particularly prominent.

Shaded habitats beneath urban forests supported a distinctive assemblage of insect-pollinated wild plant species (Figure 4) that differed markedly from those recorded in more open urban habitats (Table 4). The predominance of woodland perennials reflects the particular environmental conditions of forest understories, where reduced irradiance, lower temperature fluctuations and higher soil moisture favour shade-tolerant species adapted to relatively stable ecological conditions [36].

The dominance of hemicryptophytes and geophytes is consistent with the ecological strategies typically observed in temperate deciduous woodlands. Many geophytes complete their reproductive cycle before canopy closure, taking advantage of the high light availability occurring during early spring. This phenological adaptation allows woodland species to provide abundant nectar and pollen resources during a period when floral availability remains relatively limited in many other habitats [37].

The prolonged flowering period observed from March to October considerably enhanced the ecological value of shaded habitats. In particular, early-flowering species such as *Romulea bulbocodium*, *Scilla bifolia*, *Muscari botryoides* and the three *Allium* species constitute valuable food resources immediately after winter, whereas *Hedera helix* plays an equally important ecological role by extending nectar and pollen availability into late autumn, when few other flowering species remain available [38].

The exceptionally high visitation rates recorded for *Ajuga reptans* and *Hedera helix* indicate that these species represent important floral resources within shaded urban habitats. Their abundant nectar production and high attractiveness to pollinating insects suggest that they contribute disproportionately to sustaining pollinator populations during periods of seasonal resource limitation [39]. 

The predominance of honey bees, bumblebees and solitary bees further demonstrates that woodland understories support functionally diverse pollinator communities. Bumblebees were particularly associated with several perennial woodland herbs, whereas solitary bees predominated on numerous early-flowering species, highlighting the importance of floral diversity and phenological complementarity in maintaining resilient pollination networks within shaded environments [40].

The present findings demonstrate that urban forest understories represent valuable components of the urban green infrastructure. Beyond their recognized contribution to habitat diversity, these shaded environments provide essential floral resources during both early spring and late autumn, complementing the flowering dynamics of more open urban habitats and strengthening the temporal continuity of food resources available to pollinating insects [41].

### 3.5. Urban Wetlands

**Figure 5:**
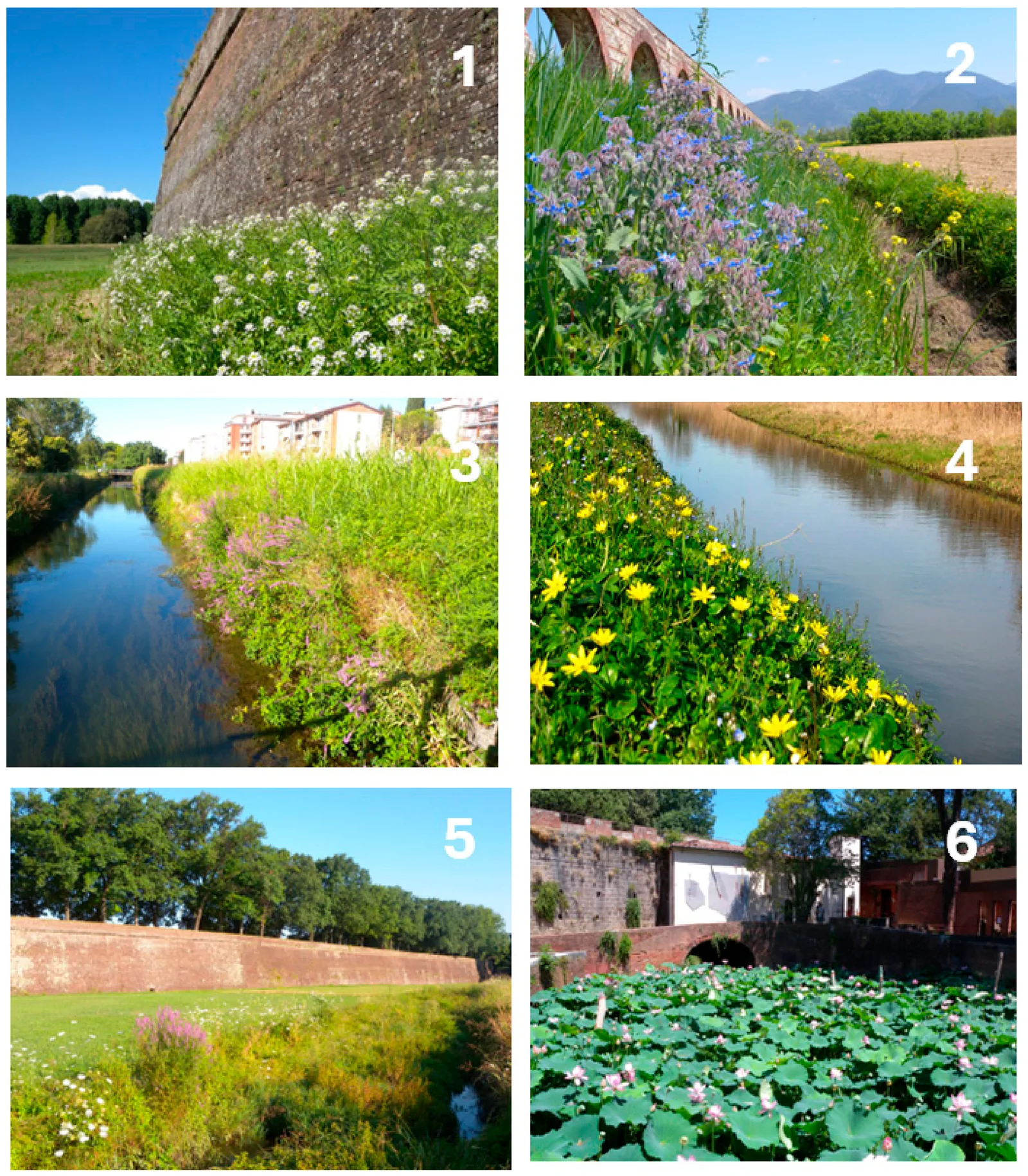
Wetland habitats where insect-pollinated plant species were recorded across the three urban ecosystems studied ((**1**,**5**) Lucca; (**2**,**3**,**6**) Pisa; (**4**) Livorno). *Berula erecta*, *Borago officinalis*, *Ranunculus ficaria*, and *Nelumbo nucifera* are shown in panels (**1**), (**2**), (**4**), and (**6**), respectively.

Urban wetlands (Figure 5) supported a highly distinctive assemblage of insect-pollinated wild plant species (Table 5), confirming that these habitats represent unique biodiversity reservoirs within the urban matrix. Their particular hydrological conditions favour specialized plant communities that differ markedly from those occurring in drier urban environments, thereby increasing habitat heterogeneity and contributing substantially to overall urban biodiversity [42].

The predominance of hemicryptophytes and geophytes reflects the relatively stable moisture regime characteristic of wetland ecosystems, whereas the occurrence of hydrophytes and hygrophilous species further emphasizes the ecological uniqueness of these habitats. Such structural and taxonomic diversity enhances habitat complexity and provides a broad range of ecological niches for pollinating insects [43].

The prolonged flowering period, extending from early spring until late summer, considerably increased the ecological value of urban wetlands. Early-flowering species such as *Ranunculus ficaria*, *Caltha palustris*, *Borago officinalis* and *Cerinthe major* provided essential nectar and pollen immediately after winter, whereas *Lythrum salicaria*, *Mentha suaveolens*, *Polygonum lapathifolium* and *Pulicaria dysenterica* ensured abundant floral resources during late summer, when nectar availability often declines in many other urban habitats [44].

*Nelumbo nucifera*, although cultivated as an ornamental species in urban ponds, *Nelumbo nucifera* was included because it was flowering during the survey and was regularly visited by insect pollinators. Its occurrence in the surveyed urban wetland was unexpected and even attracted attention in the local press. Although its exact origin remains unknown, its presence is clearly attributable to anthropochorous introduction rather than spontaneous colonization.

Several wetland species, particularly *Borago officinalis*, *Lythrum salicaria*, *Helianthus tuberosus*, *Amorpha fruticosa* and *Polygonum lapathifolium*, exhibited exceptionally high pollinator visitation rates, indicating that they function as key floral resources within these ecosystems. The conservation of such highly attractive species could therefore enhance the ecological functionality of urban wetlands and strengthen pollination services across the wider urban landscape [45]. Although several spontaneous flowering plants recorded in this study provide valuable nectar and pollen resources for urban pollinators, their management should consider their biogeographical status. Native and non-invasive naturalized species can substantially contribute to maintaining pollinator diversity and should be preferentially conserved within urban green spaces. In contrast, invasive alien species, such as *Amorpha fruticosa*, *Phytolacca americana*, *Helianthus tuberosus*, and *Oxalis corniculata*, despite their attractiveness to pollinating insects, may reduce native plant diversity through competitive exclusion and, over time, negatively affect the diversity and stability of pollinator communities. Therefore, the promotion of spontaneous urban vegetation should focus on native and non-invasive species, whereas invasive alien plants should continue to be controlled in accordance with current conservation regulations.

The balanced representation of honey bees, solitary bees and bumblebees demonstrates that wetlands support functionally diverse pollinator assemblages. In addition, the occurrence of hoverflies and tachinid flies as predominant visitors on several Apiaceae highlights the contribution of wetlands to maintaining a broad diversity of pollinating insects beyond bees alone. Such functional diversity is widely recognized as an important determinant of pollination stability and ecosystem resilience [46].

The present findings demonstrate that urban wetlands represent key ecological refuges for pollinating insects. Their combination of high floristic diversity, prolonged flowering and abundant floral resources during periods of seasonal scarcity makes these habitats particularly valuable for sustaining pollinator populations and enhancing the ecological connectivity of urban green infrastructures [47].

The conservation and management of spontaneous urban vegetation should be based on a selective, evidence-based approach. Although many spontaneous flowering plants recorded in this study represent valuable nectar and pollen resources for pollinators, their management should take into account their biogeographical status. Native and non-invasive naturalized species can substantially contribute to sustaining urban pollinator communities and should be preferentially conserved where appropriate. In contrast, invasive alien species, despite their attractiveness to pollinating insects, should not be promoted because of their potential negative impacts on native biodiversity and ecosystem functioning. For example, *Amorpha fruticosa*, although frequently visited by pollinators, is recognized as an invasive alien species of Union concern and should therefore continue to be managed according to current conservation regulations.

The five urban habitat types surveyed provided complementary floral resources for insect pollinators throughout the flowering season. While each habitat was characterized by a distinct assemblage of spontaneous plant species and pollinator visitors, their combined contribution ensured a continuous supply of nectar and pollen resources, supporting diverse pollinator guilds across the urban landscape. These findings emphasize that maintaining a mosaic of urban habitats with spontaneous flowering vegetation, rather than focusing on individual habitat types, is likely to maximize ecological benefits for pollinator conservation in Mediterranean cities.

In summary, despite these limitations, the present three-year survey provides a robust overview of the diversity of spontaneous flowering plants and their associated pollinator communities across representative Mediterranean urban habitats. The findings offer useful information to support evidence-based urban biodiversity conservation and the sustainable management of spontaneous urban vegetation. Some limitations of this study should be acknowledged. First, the dataset is primarily descriptive and was designed to characterize patterns of spontaneous urban flora and pollinator visitation across different Mediterranean urban habitat types rather than to establish causal relationships. Second, flower-visiting insects were classified into broad taxonomic groups, and species-level identification was not systematically performed for all pollinator taxa; consequently, the study does not capture the full taxonomic diversity of the pollinator community. Third, data from Pisa, Livorno, and Lucca were pooled according to habitat type because the study was not designed to test differences among cities. The three cities were used as a broader geographical sampling framework to characterize spontaneous urban flora and pollinator visitation under Mediterranean conditions in Tuscany. Therefore, potential city-specific effects cannot be assessed from the present dataset. These limitations should be considered when interpreting the results and provide directions for future studies incorporating finer taxonomic resolution and sampling designs specifically structured to evaluate spatial variation among cities.

### 3.6. Food Webs and Biodiversity in Urban Ecosystems

**Figure 6:**
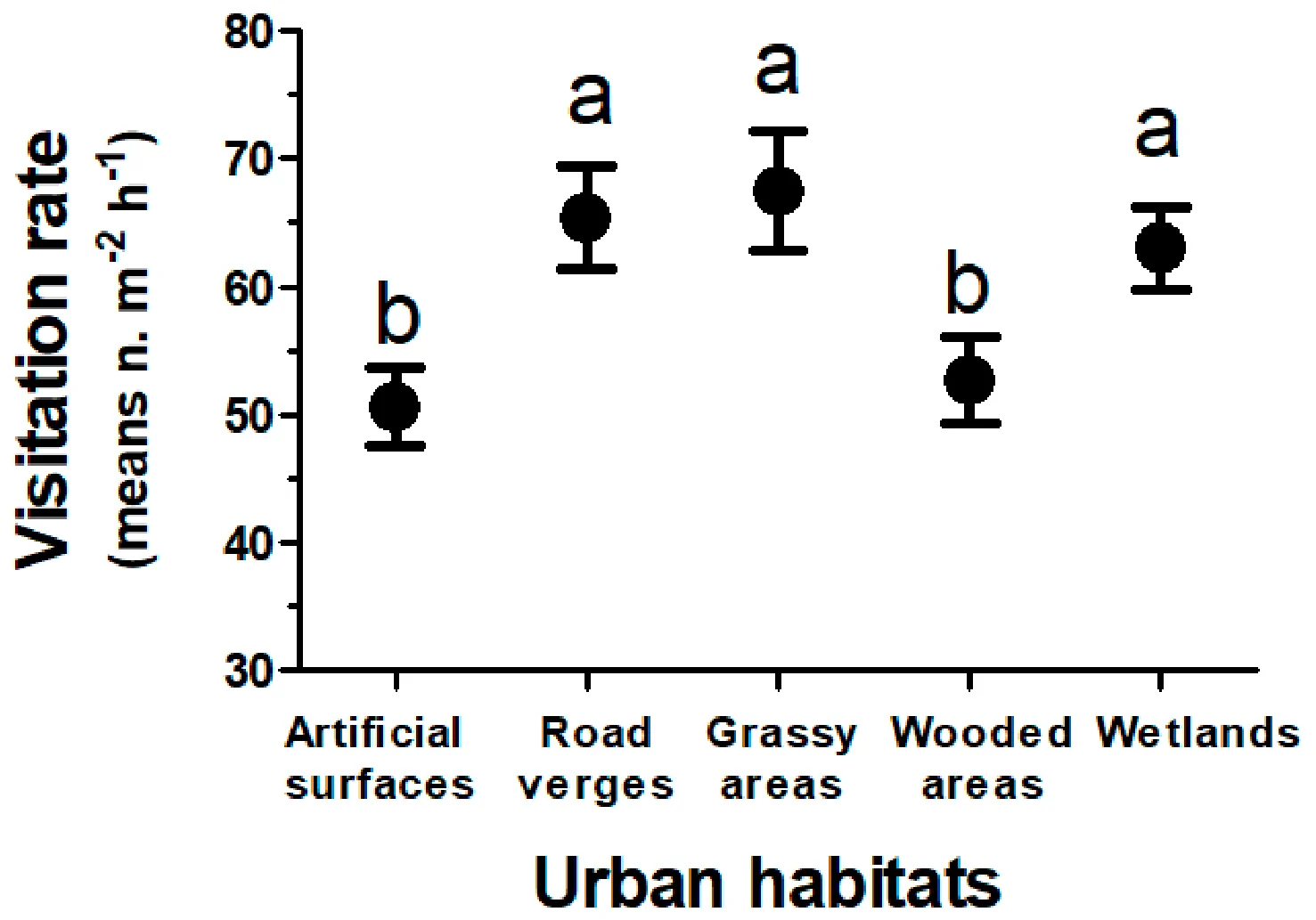
Pollinator visitation rate (mean values across the respective plant species, expressed as the number of visits m^−2^ h^−1^) recorded in the different urban habitats (artificial surfaces, road verges, grassy areas, wooded areas, and wetlands). Vertical bars indicate ± standard errors of the means (SE). Means followed by the same letter are not significantly different at *p* < 0.05.

**Figure 7:**
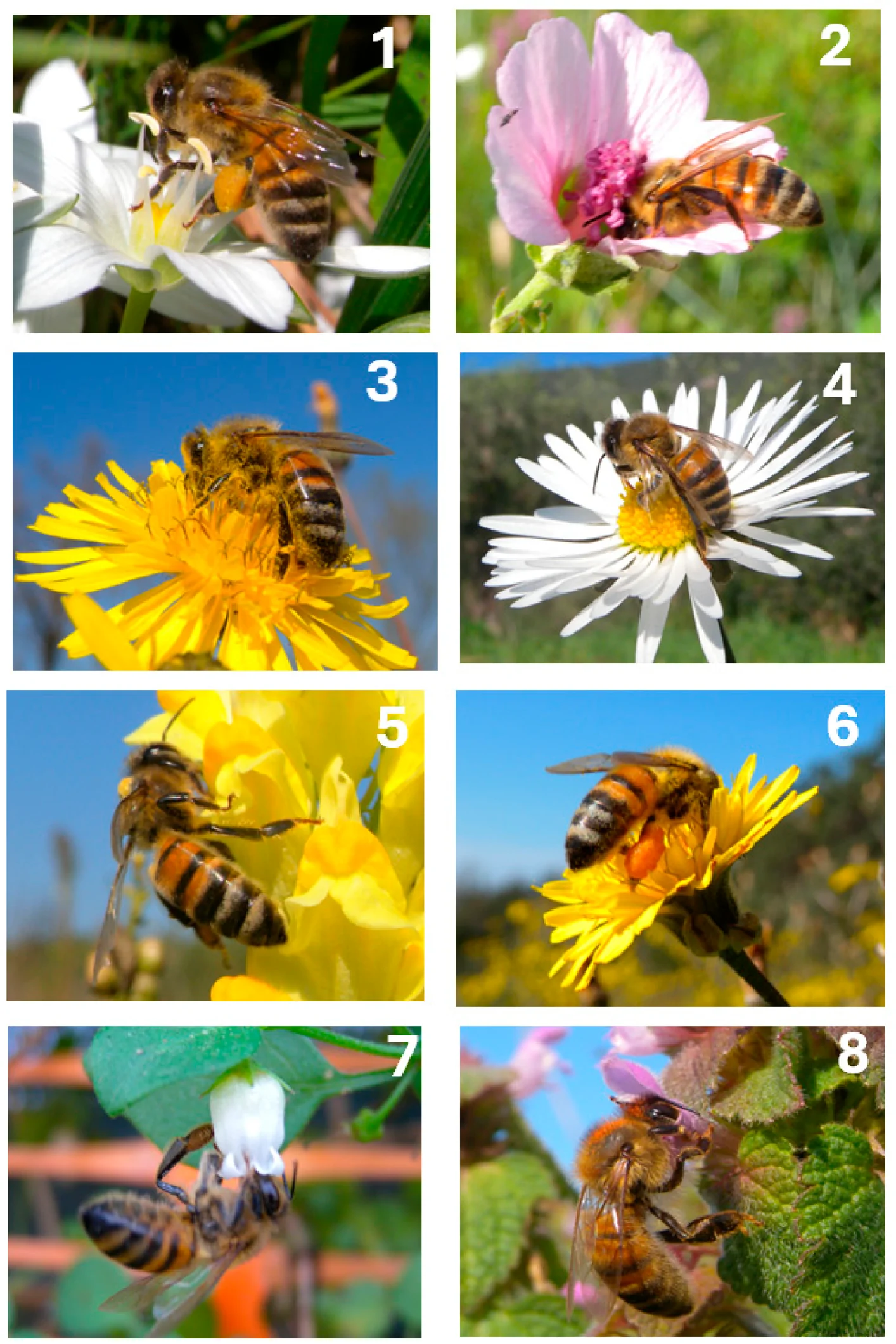
Honey bees observed visiting the flowers of ((**1**) *Ornithogalum umbellatum*, (**2**) *Althaea cannabina*, (**3**) *Crepis sancta*, (**4**) *Bellis perennis*, (**5**) *Linaria vulgaris*, (**6**) *Reichardia picroides*, (**7**) *Salpichroa origanifolia*, (**8**) *Lamium purpureum*) in the different urban habitats of the three study cities (Pisa, Livorno, and Lucca).

**Figure 8:**
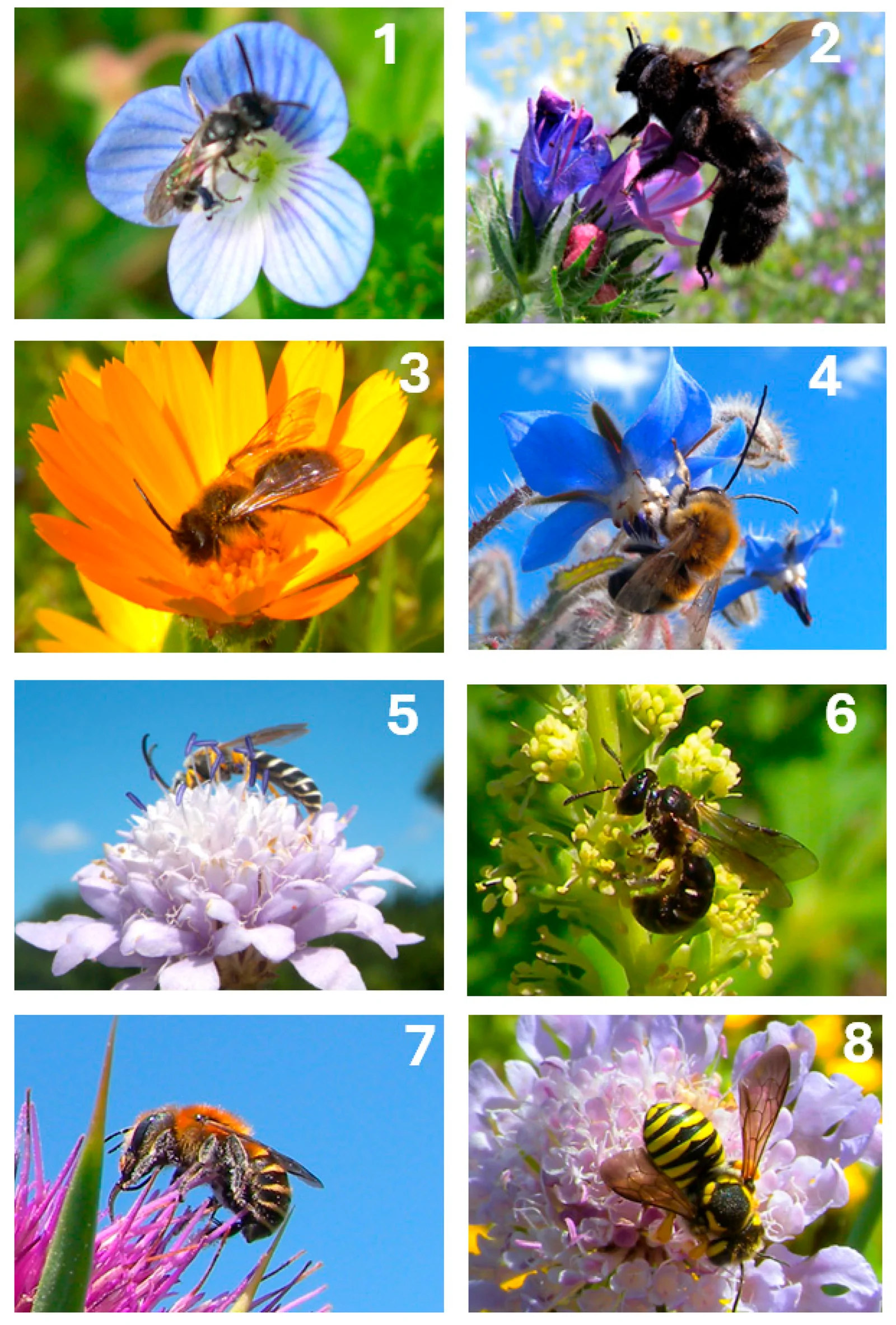
Solitary bees observed visiting the flowers of (**1**) *Veronica persica*, (**2**) *Echium vulgare*, (**3**) *Calendula arvensis*, (**4**) *Borago officinalis*, (**5**) *Cephalaria transsylvanica*, (**6**) *Reseda alba*, (**7**) *Silybum marianum*, (**8**) *Scabiosa columbaria* in the different urban habitats of the three study cities (Pisa, Livorno, and Lucca).

**Figure 9:**
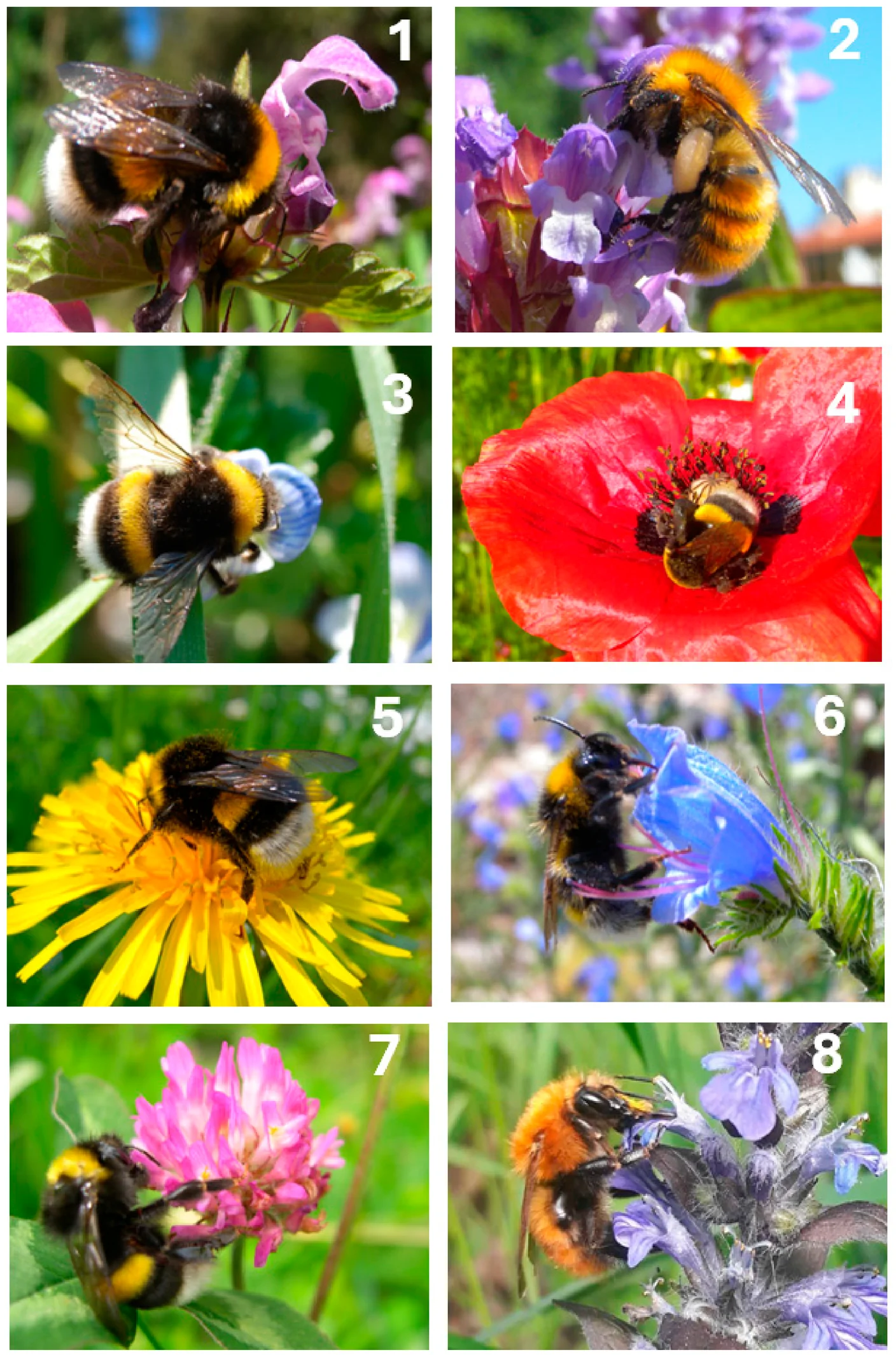
Bumblebees observed visiting the flowers of (**1**) *Lamium maculatum*, (**2**) *Prunella vulgaris*, (**3**) *Veronica persica*, (**4**) *Papaver rhoeas*, (**5**) *Taraxacum officinale*, (**6**) *Echium vulgare*, (**7**) *Hedysarum coronarium*, (**8**) *Ajuga reptans* in the different urban habitats of the three study cities (Pisa, Livorno, and Lucca).

**Figure 10:**
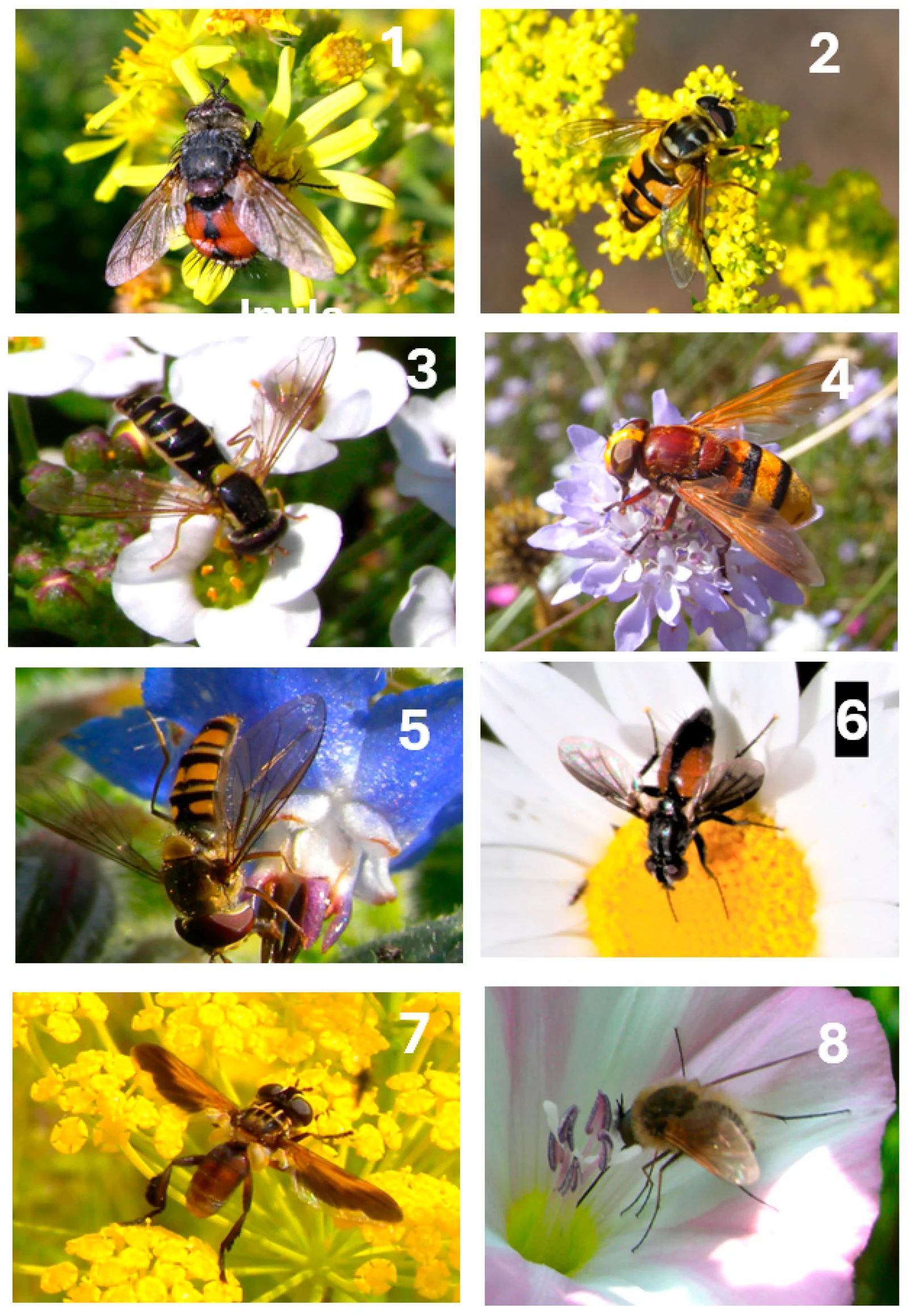
Dipterans (hoverflies: (**2**–**5**); tachinid flies: (**1**,**6**,**7**); bee flies: (**8**) observed visiting the flowers of (**1**) *Inula viscosa*, (**2**) *Galium verum*, (**3**) *Lobularia maritima*, (**4**) *Cephalaria transsylvanica*, (**5**) *Borago officinalis*, (**6**) *Bellis perennis*, (**7**) *Foeniculum vulgare*, (**8**) *Convolvulus arvensis* in the different urban habitats of the three study cities (Pisa, Livorno, and Lucca).

**Figure 11:**
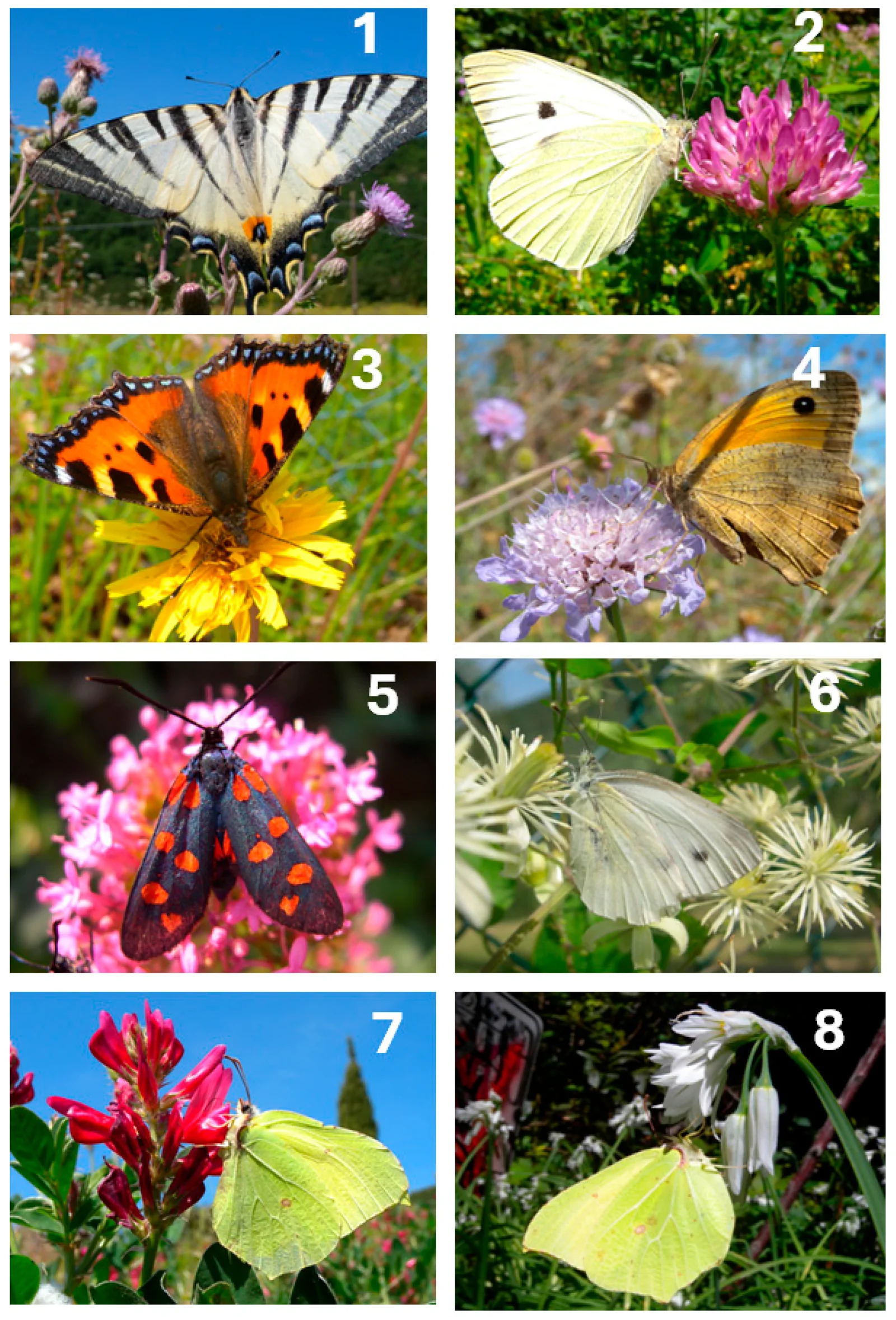
Lepidopterans observed visiting the flowers of (**1**) *Cirsium arvense*, (**2**) *Trifolium pratense*, (**3**) *Hyoseris radiata*, (**4**) *Cephalaria transsylvanica*, (**5**) *Centranthus ruber*, (**6**) *Clematis vitalba*, (**7**) *Hedysarum coronarium*, (**8**) *Allium triquetrum* in the different urban habitats of the three study cities (Pisa, Livorno, and Lucca).

The foraging activity of pollinators was recorded across the different urban habitats (Figure 6) and included honey bees (Figure 7), solitary bees (Figure 8), bumblebees (Figure 9), dipterans (Figure 10), and lepidopterans (Figure 11).

Pollinator activity differed significantly among the investigated urban habitats, with grassy areas, roadside verges and wetlands (Table 1, Table 2, Table 3, Table 4, Table 5 and Table S1) supporting consistently higher visitation rates than artificial surfaces and wooded habitats. These results indicate that open herbaceous habitats provide more favourable foraging conditions for pollinating insects, probably because of their greater floral abundance, higher flower density and prolonged seasonal availability of nectar and pollen resources [48].

The lower visitation rates recorded in artificial surfaces and shaded woodland habitats do not necessarily indicate a reduced ecological importance of these environments. Instead, these habitats appear to complement the more productive herbaceous communities by providing floral resources during specific periods of the year or for particular pollinator guilds, thereby increasing habitat heterogeneity and strengthening the temporal continuity of resource availability within the urban landscape [13].

The photographic documentation confirms the remarkable ecological plasticity of *Apis mellifera*, which exploited flowers belonging to numerous botanical families and occurring across a broad spectrum of urban habitats. This wide trophic niche allows honey bees to efficiently exploit the heterogeneous distribution of floral resources typical of urban environments and partly explains their success in cities [35].

Similarly, solitary bees visited a remarkably diverse array of spontaneous flowering species, highlighting the importance of urban wild flora for sustaining wild bee communities. Because many solitary bee species exhibit more restricted foraging ranges and nesting requirements than honey bees, the availability of diverse spontaneous flowering plants within urban habitats is likely to play a crucial role in maintaining their populations [49].

Bumblebees were predominantly associated with highly rewarding nectar-producing species, confirming their preference for plants offering abundant floral rewards capable of satisfying the energetic requirements of large-bodied social bees. The occurrence of these species across different urban habitats further emphasizes the contribution of spontaneous vegetation to sustaining bumblebee populations within cities [50].

Dipterans, including hoverflies, tachinid flies and bee flies, exploited a broad range of flowering species, demonstrating that spontaneous urban vegetation supports pollinator communities extending well beyond bees. Since many dipteran species also contribute to biological control through predation or parasitism during their larval stages, their conservation may provide multiple ecosystem services in addition to pollination [51].

Butterflies were associated with numerous spontaneous flowering species (Figure 11) distributed across different urban habitats, confirming the importance of continuous nectar availability for adult Lepidoptera. Because butterfly diversity is often considered a sensitive indicator of habitat quality and ecological connectivity, their widespread occurrence further highlights the conservation value of spontaneous urban vegetation [52].

**Figure 12:**
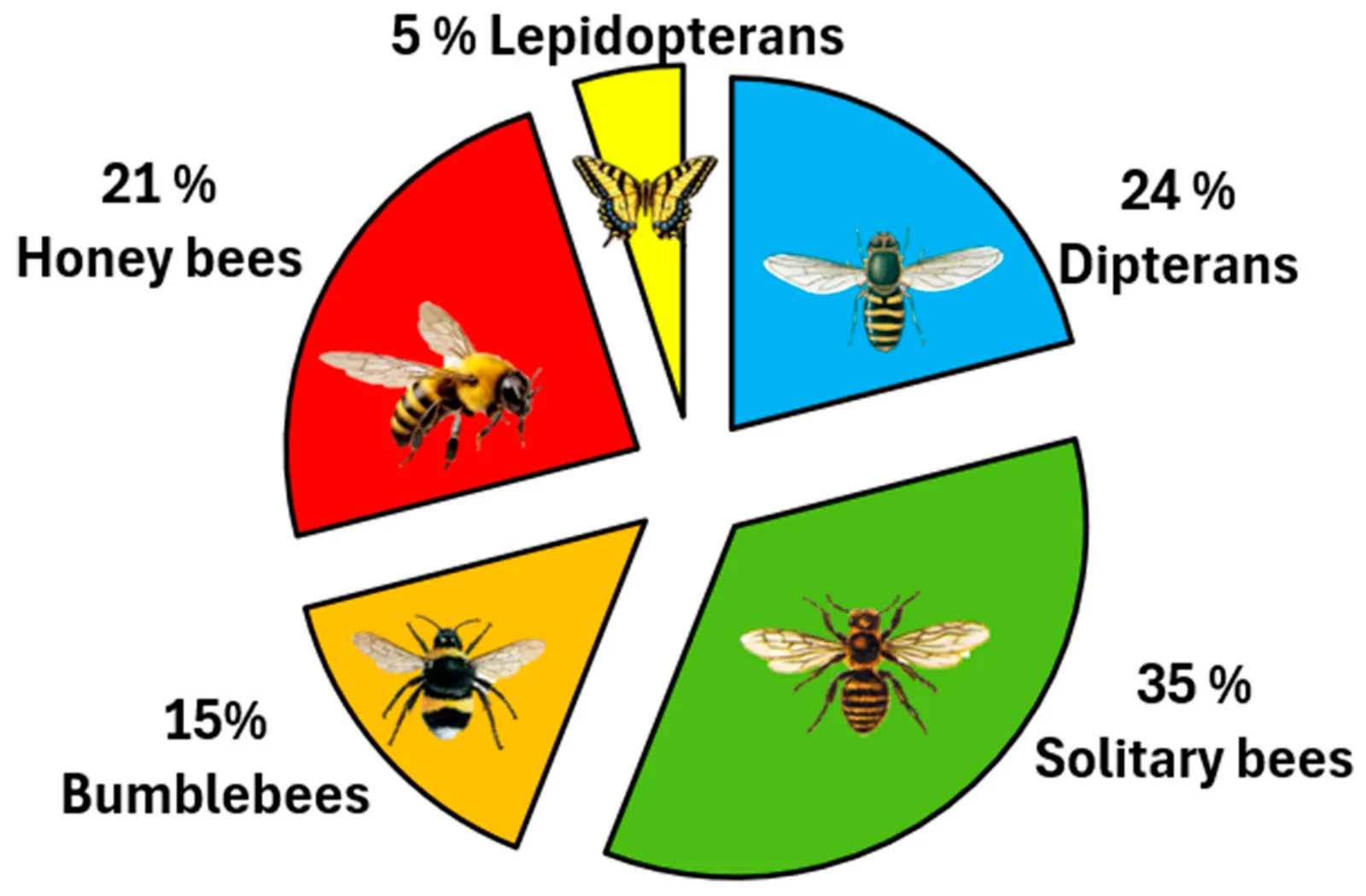
The relative abundances of the five pollinator groups were calculated as percentages of the total number of recorded visits and are presented descriptively. These findings demonstrate that spontaneous urban flora should not be regarded merely as vegetation to be controlled but rather as an important ecological component capable of enhancing biodiversity and supporting essential ecosystem services such as pollination. Consequently, urban green-space management should adopt more biodiversity-friendly practices that preserve and promote spontaneous flowering vegetation wherever compatible with public use and safety. Such an approach would contribute not only to pollinator conservation but also to improving the ecological quality, resilience, and sustainability of urban ecosystems.

The composition of the pollinator community (Figure 12) clearly demonstrates that honey bees and solitary bees constitute the dominant pollinating groups within Mediterranean urban ecosystems, whereas Diptera, bumblebees and Lepidoptera provide complementary contributions to pollination services. The coexistence of these functionally distinct pollinator guilds enhances the stability and resilience of urban pollination networks, reinforcing the ecological importance of conserving diverse spontaneous flowering vegetation across multiple urban habitat types [53].

The ecological value of roadside spontaneous vegetation, however, depends on appropriate management rather than on the absence of maintenance. Since road verges are routinely mown for safety and operational reasons, management practices should be adapted to local flowering phenology whenever feasible. Selective or delayed mowing can prolong the availability of nectar and pollen resources for pollinators while maintaining the functional requirements of urban infrastructure. Such an evidence-based approach would contribute to preserving the role of road verges within urban food webs without compromising public safety.

Future studies integrating habitat characteristics, patch size, seasonal variation, and additional environmental variables into multifactorial models, together with comparisons between spontaneous and managed ornamental vegetation, would further improve our understanding of the relative contribution of different urban plant communities to pollinator conservation.

## 4. Conclusions

Urban spontaneous vegetation plays a crucial role in supporting pollinator diversity by providing abundant and continuous floral resources across a wide range of urban habitats. The present study demonstrated that the spontaneous flora occurring in the Mediterranean cities of Pisa, Livorno, and Lucca sustains a rich pollinator community, including honey bees, solitary bees, bumblebees, Diptera, and Lepidoptera, although their relative abundance differed among habitat types. Pollinator activity was consistently higher in herbaceous habitats, such as grasslands, road verges, and wetlands, than in wooded areas and highly artificial environments, emphasizing the importance of maintaining open, flower-rich habitats within urban landscapes. The large number of insect-pollinated plant species recorded and their extended flowering periods ensure a continuous supply of nectar and pollen throughout the growing season, thereby contributing to the stability of urban plant–pollinator interactions. A further small proportion of flower visitors (less than 1%) consisted of vespids, lacewings, and beetles.

In summary, the present three-year survey demonstrates that spontaneous flowering vegetation in Mediterranean urban environments provides complementary floral resources that support diverse pollinator communities throughout the flowering season. The conservation of a heterogeneous mosaic of urban habitats rich in native and non-invasive spontaneous plant species should therefore be considered an important component of evidence-based urban biodiversity management. However, although some invasive alien plants may represent valuable nectar and pollen sources, their potential negative impacts on native plant communities and ecosystem functioning outweigh these short-term ecological benefits. Consequently, invasive species should continue to be managed according to current conservation policies, while conservation efforts should primarily promote native and non-invasive spontaneous vegetation.

## Figures and Tables

**Table 1:** Weed species occurring in urban built environments (walls, roofs, and sidewalks), with their corresponding botanical and biological traits, degree of spread, attractiveness to pollinators, and predominant interactions with pollinator groups. Pollinator visitation rates are reported as mean ± standard deviation (SD) based on nine patch-level observations per species (three independent flowering patches × three study years; *n* = 9).

Weed Species	Botanic Family ^1^	Life Cycle ^2^	Peak Flowering (Months)	Pollinator Visitation Rate (n. m^−2^ h^−1^)	Prevalent Pollinator Type ^3^
*Antirrhinum latifolium* Miller	Scrophulariaceae	Ch	May–June	43.5 ± 5.2	BB
*Antirrhinum majus* L.	Scrophulariaceae	Ch	May–July	52.8 ± 6.1	BB
*Calamintha nepeta* (L.) Savi	Lamiaceae	Ch	August–September	42.4 ± 4.7	SB
*Campanula erinus* L.	Campanulaceae	T	April–May	10.2 ± 1.5	SB
*Capparis spinosa* L.	Capparidaceae	NF	June–August	48.5 ± 5.1	SB
*Centranthus ruber* (L.) DC.	Valerianaceae	Ch	May–June	95.7 ± 10.3	L
*Cerastium glomeratum* Thuill.	Caryophyllaceae	T	March–April	39.4 ± 4.0	SF
*Cheiranthus cheiri* L.	Brassicaceae	Ch	April	47.5 ± 5.3	SB
*Crepis sancta* (L.) Bornm.	Asteraceae	T	April–May	91.4 ± 9.8	HB
*Diplotaxis tenuifolia* (L.) DC.	Brassicaceae	T	June–September	66.5 ± 7.2	HB
*Erigeron karvinskianus* DC.	Asteraceae	H	May–October	28.4 ± 3.3	HB
*Fumaria capreolata* L.	Papaveraceae	T	March–April	37.1 ± 4.3	SB
*Helichrysum italicum* (Roth) G.Don	Asteraceae	Ch	June–July	20.6 ± 2.9	HB
*Inula viscosa* (L.) Aiton	Asteraceae	Ch	September	104.9 ± 12.5	SB
*Lobularia maritima* (L.) Desv.	Brassicaceae	H	April–June	60.4 ± 7.3	SF
*Oxalis corniculata* L.	Oxalidaceae	H	June–September	56.2 ± 6.6	SB
*Portulaca oleracea* L.	Portulacaceae	T	July–August	20.3 ± 2.8	SB
*Reichardia picroides* (L.) Roth	Asteraceae	H	April–June	86.4 ± 9.5	SB
*Reseda alba* L.	Resedaceae	H	May–July	50.4 ± 5.9	SB
*Sedum rupestre* L.	Crassulaceae	Ch	July	80.2 ± 8.7	SF
*Sonchus asper* (L.) Hill	Asteraceae	T	April–June	50.3 ± 6.2	HB
*Sonchus oleraceus* L.	Asteraceae	T	April–June	45.7 ± 5.0	HB
*Sonchus tenerrimus* L.	Asteraceae	T	April–May	42 ± 4.7	HB
*Trifolium campestre* Schreb.	Fabaceae	T	May–June	34.8 ± 3.9	BB
*Verbascum sinuatum* L.	Scrophulariaceae	H	June–July	48.5 ± 5.2	BB
*Veronica cymbalaria* Board	Scrophulariaceae	T	March–April	20.6 ± 3.1	SB

^1^ Cronquist classification system. ^2^ T = therophyte, H = hemicryptophyte, G = Geophyte, Ch = Chamaephyte, NF = nanophanerophyte. ^3^ HB = honeybees, SB = solitary bees, BB = bumblebees, SF = Syrphid flies, BF = bombyliid flies, L = lepidoptera.

**Table 2:** Weed species occurring in roadside verges, with their corresponding botanical and biological traits, degree of spread, attractiveness to pollinators, and predominant interactions with pollinator groups. Pollinator visitation rates are reported as mean ± standard deviation (SD) based on nine patch-level observations per species (three independent flowering patches × three study years; *n* = 9).

Weed Species	Botanic Family ^1^	Life Cycle ^2^	Peak Flowering (Months)	Pollinator Visitation Rate (n. m^−2^ h^−1^)	Prevalent Pollinator Type ^3^
*Allium schoenoprasum* L.	Liliaceae	G	May–June	68.5 ± 7.3	SB
*Althaea cannabina* L.	Malvaceae	H	July–August	64.1 ± 6.7	HB
*Anagallis arvensis* L.	Primulaceae	T	April–May	35.2 ± 3.9	SB
*Anchusa hybrida Ten.*	Boraginaceae	H	April–May	45.8 ± 5.1	HB
*Calendula arvensis* (Vaill.) L.	Astraceae	T	March–April	58.3 ± 6.3	SB
*Cephalaria transsylvanica* (L.) Sch.	Dipsacaceae	T	June–July	135.3 ± 14.5	SB
*Cichorium intybus* L.	Asteraceae	H	July–August	89.1 ± 9.3	SB
*Convolvulus arvensis* L.	Convolvulaceae	G	June–August	58.4 ± 6.1	SF
*Crepis leontodontoides* All.	Asteraceae	T	May–June	43.7 ± 4.8	HB
*Erodium cicutarium* (L.) L’Hér.	Geraniaceae	T	April–May	25.6 ± 2.9	SF
*Foeniculum vulgare* Mill.	Apiaceae	H	July–August	52.4 ± 6.0	TF
*Galium verum* L.	Rubiaceae	H	June–July	26.4 ± 3.1	SF
*Hedysarum coronarium* L.	Fabaceae	T	April–May	108.4 ± 11.5	HB
*Hyoseris radiata* L.	Asteraceae	H	April–May	87.3 ± 9.3	SB
*Lotus corniculatus* L.	Fabaceae	H	May–July	97.3 ± 9.6	HB
*Malva neglecta* Wallr.	Malvaceae	H	May–July	78.5 ± 8.2	SB
*Malva sylvestris* L.	Malvaceae	H	May–June	86.4 ± 9.4	HB
*Myosotis ramosissima* Rochel	Boraginaceae	T	April–May	23.2 ± 2.7	BF
*Oxalis articulata* Savigny	Oxalidaceae	G	June–September	45.8 ± 4.9	SB
*Papaver rhoeas* L.	Papaveraceae	T	April–May	77.4 ± 8.1	BB
*Potentilla reptans* L.	Rosaceae	G	June–August	30.5 ± 3.6	SB
*Poterium sanguisorba* L.	Rosaceae	H	May–June	20.6 ± 2.8	SB
*Sedum album* L.	Crassulaceae	Ch	June–July	67.4 ± 7.3	HB
*Salvia verbenaca* L.	Lamiaceae	H	April–May	86.4 ± 9.4	HB
*Scabiosa columbaria* L.	Dipsacaceae	H	June–July	130.5 ± 14.3	SB
*Scolymus hispanicus* L.	Asteraceae	H	June–July	60.2 ± 7.2	SB
*Trifolium repens* L.	Fabaceae	H	May–July	95.8 ± 10.2	HB
*Urospermum dalechampii* (L.) Sch.	Asteraceae	H	April–May	45.3 ± 5.2	SB
*Verbascum sinuatum* L.	Scrophulariaceae	H	June–July	53.8 ± 5.8	SB

^1^ Cronquist classification system. ^2^ T = therophyte, H = hemicryptophyte, G = Geophyte, Ch = Chamaephyte, NF = nanophanerophyte. ^3^ HB = honeybees, SB = solitary bees, BB = bumblebees, SF = Syrphid flies, BF = bombyliid flies, TF = Tachinid flies, L = lepidoptera.

**Table 3:** Weed species occurring in urban herbaceous patches and grasslands, with their corresponding botanical and biological traits, degree of spread, attractiveness to pollinators, and predominant interactions with pollinator groups. Pollinator visitation rates are reported as mean ± standard deviation (SD) based on nine patch-level observations per species (three independent flowering patches × three study years; *n* = 9).

Weed Species	Botanic Family ^1^	Life Cycle ^2^	Peak Flowering (Months)	Pollinator Visitation Rate (n. m^−2^ h^−1^)	Prevalent Pollinator Type ^3^
*Bellis perennis* L.	Asteraceae	H	April–May	70.4 ± 8.2	HB
*Lavatera punctata* All.	Malvaceae	T	May–June	78.5 ± 8.6	HB
*Centaurium erythraea* Rafn	Gentianaceae	T	June–July	32.1 ± 3.9	BF
*Cirsium arvense* (L.) Scop.	Asteraceae	G	June–July	107.3 ± 12.7	HB
*Convolvulus arvensis* L.	Convolvulaceae	G	June–August	43.7 ± 4.8	SF
*Crepis leontodontoides* All.	Asteraceae	H	May–June	78.5 ± 8.2	HB
*Crepis vesicaria*	Asteraceae	T	April–May	98.2 ± 10.4	SB
*Daucus carota* L.	Apiaceae	H	June–July	70.0 ± 7.7	SF
*Echium vulgare* L.	Boraginaceae	H	June–July	95.3 ± 9.8	BB
*Fumaria officinalis* L.	Papaveraceae	T	April–May	32.0 ± 3.9	SB
*Geranium molle* L.	Geraniaceae	T	April–May	34.8 ± 4.3	SB
*Hypericum perforatum* L.	Hypericaceae	H	June–July	53.2 ± 5.8	SB
*Hypochaeris radicata* L.	Asteraceae	H	June–September	88.7 ± 9.4	HB
*Lamium amplexicaule* L.	Lamiaceae	T	March–April	90.4 ± 9.7	HB
*Lamium purpureum* L.	Lamiaceae	T	March–April	58.2 ± 6.5	HB
*Muscari botrioides* (L.) Mill.	Liliaceae	G	March–April	80.4 ± 8.6	HB
*Ornithogalum umbellatum* L.	Liliaceae	G	April	97.3 ± 10.5	HB
*Picris echioides* L.	Asteraceae	T	July–August	89.5 ± 9.5	SB
*Picris hieracioides* L.	Asteraceae	H	July–August	77.3 ± 8.4	SB
*Sherardia arvensis* L.	Rubiaceae	T	April–May	28.5 ± 3.8	BF
*Silene latifolia* Poir.	Caryophyllaceae	H	June–July	40.6 ± 4.9	L
*Stellaria media* (L.) Vill.	Caryophyllaceae	T	March–May	30.7 ± 3.6	SB
*Taraxacum officinale* Weber	Asteraceae	H	April–May	90.6 ± 9.9	BB
*Torilis arvensis* (Huds.) Link	Apiaceae	T	June–July	78.4 ± 8.4	SF
*Tragopogon pratensis* L.	Asteraceae	H	May–June	76.5 ± 8.1	SB
*Trifolium pratense* L.	Fabaceae	H	May–June	108.4 ± 12.5	BB
*Verbascum blattaria* L.	Scrophulariaceae	H	June–July	45.4 ± 4.9	SB
*Verbena officinalis* L.	Verbenaceae	H	July–August	32.1 ± 3.7	BF
*Veronica persica* Poir.	Scrophulariaceae	T	March–April	50.6 ± 5.8	SB

^1^ Cronquist classification system. ^2^ T = therophyte, H = hemicryptophyte, G = Geophyte, Ch = Chamaephyte, NF = nanophanerophyte. ^3^ HB = honeybees, SB = solitary bees, BB = bumblebees, SF = Syrphid flies, BF = bombyliid flies, L = lepidoptera.

**Table 4:** Weed species occurring in areas shaded by urban forests, with their corresponding botanical and biological traits, degree of spread, attractiveness to pollinators, and predominant interactions with pollinator groups. Pollinator visitation rates are reported as mean ± standard deviation (SD) based on nine patch-level observations per species (three independent flowering patches × three study years; *n* = 9).

Weed Species	Botanic Family ^1^	Life Cycle ^2^	Peak Flowering (Months)	Pollinator Visitation Rate (n. m^−2^ h^−1^)	Prevalent Pollinator Type ^3^
*Acanthus mollis* L.	Acanthaceae	H	May–June	30.7 ± 3.3	BB
*Ajuga reptans* L.	Lamiaceae	H	April–May	112.1 ± 14.2	BB
*Alliaria petiolata* M. Bieb.	Brassicaceae	H	April–May	50.3 ± 5.7	SB
*Allium neapolitanum* Cyr.	Liliaceae	G	April	87.4 ± 9.2	HB
*Allium roseum* L.	Liliaceae	G	April–May	85.2 ± 8.9	HB
*Allium triquetrum* L.	Liliaceae	G	March–April	75.6 ± 8.0	HB
*Chelidonium majus* L.	Papaveraceae	H	April–May	42.3 ± 4.7	SB
*Clematis vitalba* L.	Ranunculaceae	P	June–July	58.2 ± 6.4	HB
*Clinopodium vulgare* L.	Lamiaceae	H	July–August	50.4 ± 5.6	BB
*Cymbalaria muralis* P. Gaer. & al.	Scrophulariaceae	H	April–May	18.3 ± 2.2	SB
*Geranium robertianum* L.	Geraniaceae	H	May–June	21.2 ± 2.8	BB
*Glechoma hederacea L.*	Lamiaceae	H	April–May	34.5 ± 3.9	SB
*Hedera helix* L.	Araliaceae	P	September–October	108.4 ± 11.3	HB
*Lamium maculatum* L.	Lamiaceae	H	April–May	76.2 ± 7.9	BB
*Lunaria annua* L.	Brassicaceae	H	May	69. 4 ± 7.3	HB
*Muscari botrioides* (L.) Mill.	Liliaceae	G	March–April	88.5 ± 9.2	SB
*Phytolacca americana* L.	Phytolaccaceae	G	July–August	24.6 ± 2.9	HB
*Prunella vulgaris* L.	Lamiaceae	H	June–July	71.4 ± 7.7	HB
*Romulea bulbocodium* L.	Iridaceae	G	March	15.3 ± 1.9	SB
*Rubus ulmifolius* Schott	Rosaceae	P	May–June	69.4 ± 7.6	HB
*Salpichroa origanifolia* (Lam.) Baill.	Solanaceae	Ch	July–September	36.2 ± 4.2	HB
*Scilla bifolia* L.	Liliaceae	G	March–April	34.1 ± 3.8	SB
*Solanum dulcamara* L.	Solanaceae	NP	July–August	23.5 ± 2.7	BB
*Solanum nigrum* L.	Solanaceae	T	July–August	24.1 ± 2.8	SB
*Symphytum officinale* L.	Boraginaceae	G	June–July	24.6 ± 2.7	BB
*Valerianella locusta* L.	Valerianaceae	T	April–May	14.2 ± 1.8	BF
*Vinca major* L.	Apocynaceae	Ch	April–May	33.5 ± 3.9	BB

^1^ Cronquist classification system. ^2^ T = therophyte, H = hemicryptophyte, G = Geophyte, Ch = Chamaephyte, NF = nanophanerophyte. ^3^ HB = honeybees, SB = solitary bees, BB = bumblebees, SF = Syrphid flies, BF = bombyliid flies.

**Table 5:** Weed species occurring in urban wetlands, with their corresponding botanical and biological traits, degree of spread, attractiveness to pollinators, and predominant interactions with pollinator groups. Pollinator visitation rates are reported as mean ± standard deviation (SD) based on nine patch-level observations per species (three independent flowering patches × three study years; *n* = 9).

Weed Species	Botanic Family ^1^	Life Cycle ^2^	Peak Flowering (Months)	Pollinator Visitation Rate (n. m^−2^ h^−1^)	Prevalent Pollinator Type ^3^
*Amorpha fruticosa* L.	Fabaceae	NP	June–July	88.4 ± 9.3	HB
*Apium nodiflorum* (L,) Lag.	Apiaceae	H	July–August	65.3 ± 7.2	TF
*Berula erecta* (Huds.) Coville	Apiaceae	G	June–July	45.7 ± 5.0	SF
*Borago officinalis* L.	Boraginaceae	T	April–May	125.6 ± 15.2	HB
*Caltha palustris* L.	Ranunculaceae	H	April–May	43.1 ± 4.8	SB
*Cerinthe major* L.	Boraginaceae	T	April–May	70.5 ± 8.3	HB
*Epilobium hirsutum* L.	Onagraceae	H	July–August	68.4 ± 7.5	BB
*Epilobium parviflorum* Schreb.	Onagraceae	H	July–August	55.3 ± 6.1	BB
*Helianthus tuberosus* L.	Asteraceae	G	August–September	93.4 ± 9.8	HB
*Iris pseudacorus* L.	Iridaceae	G	May	68.3 ± 7.4	BB
*Lepidium draba* L.	Brassicaceae	G	April–May	55.2 ± 5.9	SB
*Linaria vulgaris* Mill.	Scrophulariaceae	H	June–July	68.4 ± 7.3	HB
*Ludwigia peploides* (H.B.K.) Raven	Onagraceae	H	July–August	44.5 ± 4.8	HB
*Lythrum salicaria* L.	Lythraceae	H	July–August	118.4 ± 13.6	BB
*Mentha suaveolens* Ehrh.	Lamiaceae	H	July–August	64.2 ± 6.7	SB
*Nasturtium officinale* W.T.Aiton	Brassicaceae	H	May–June	45.3 ± 5.2	SF
*Nelumbo nucifera* Gaertn.	Nelumbonaceae	I	July–August	33.7 ± 3.6	HB
*Oxalis articulata* Savigny	Oxalidaceae	G	June–September	46.2 ± 5.0	SB
*Polygonum lapathifolium* L.	Polygonaceae	T	July–August	86.4 ± 9.3	HB
*Pulicaria dysenterica* (L.) Bernh.	Asteraceae	H	August–September	52.2 ± 5.8	SB
*Ranunculus ficaria* L.	Ranunculaceae	G	March–April	45.6 ± 4.9	SB
*Ranunculus repens* L.	Ranunculaceae	H	May–June	58.7 ± 6.6	SB
*Silybum marianum* (L.) Gaertn.	Asteraceae	H	May–June	78.4 ± 8.4	HB
*Symphytum bulbosum* K. F. Sch.	Boraginaceae	G	April	20.5 ± 2.6	BB
*Thalictrum flavum* L.	Ranunculaceae	H	July–August	32.6 ± 3.8	SB
*Veronica anagallis-aquatica* L.	Scrophulariaceae	H	July–August	38.7 ± 4.5	SB

^1^ Cronquist classification system. ^2^ T = therophyte, H = hemicryptophyte, G = Geophyte, Ch = Chamaephyte, NF = nanophanerophyte, I = hydrophyte. ^3^ HB = honeybees, SB = solitary bees, BB = bumblebees, SF = Syrphid flies, BF = bombyliid flies, TF = Tachinid flies.

## Data Availability

The original contributions presented in this study are included in the article/Supplementary Materials. Further inquiries can be directed to the corresponding author.

## Supplementary Materials

The following supporting information can be downloaded at: https://www.mdpi.com/article/10.3390/plants15182765/s1. Table S1. Secondary and tertiary pollinator groups ^1^ (the predominant groups are shown in Table 1, Table 2, Table 3, Table 4 and Table 5) recorded on weed species growing in different ecological niches within the urban environment during the three-year study.
