# Supplementary material for: Urban Weeds: Supporters of Pollinator Biodiversity in Urban Landscapes
Source: Plants (Basel). 2026 Sep 9;15(18):2765. doi: 10.3390/plants15182765 (PMC13610018; doi:10.3390/plants15182765)
Supplement: Supplementary file 1 [file plants-15-02765-s001.zip › plants-4475865-supplementary.pdf]

Table S1. Secondary and tertiary pollinator groups[1] (the predominant groups are shown in Tables 1–5) recorded on weed species growing in different ecological niches within the urban environment during the three-year study.

Weed Species	Secondary Pollinator Type	Tertiary pollinators Type
Acanthus mollis L.	SF	L
Ajuga reptans L.	HB	SB
Alliaria petiolata M. Bieb.	HB	SF
Allium neapolitanum Cyr.	SB	BF
Allium roseum L.	SB	L
Allium schoenoprasum L.	HB	L
Allium triquetrum L.	SB	L
Althaea cannabina L.	SB	L
Anagallis arvensis L.	SF	BF
Anchusa hybrida Ten.	SB	BF
Antirrhinum latifolium Miller	SB	HB
Antirrhinum majus L.	SB	HB
Bellis perennis L.	SF	SB
Calamintha nepeta (L.) Savi	HB	L
Calendula arvensis (Vaill.) L.	HB	L
Campanula erinus L.	BF	L
Capparis spinosa L.	HB	L
Centaurium erythraea Rafn	SB	L
Centranthus ruber (L.) DC.	SB	BF
Cephalaria transsylvanica (L.) Sch.	HB	L
Cerastium glomeratum Thuill.	TF	L
Cheiranthus cheiri L.	HB	L
Chelidonium majus L.	HB	SF
Cichorium intybus L.	HB	L
Cirsium arvense (L.) Scop.	SB	SF
Clematis vitalba L.	L	SB
Clinopodium vulgare L.	HB	SB
Convolvulus arvensis L.	BB	SB
Crepis leontodontoides All.	SB	SF
Crepis sancta (L.) Bornm.	SF	L
Crepis vesicaria	HB	L
Cymbalaria muralis P. Gaer. & al.	BF	L
Daucus carota L.	SB	HB
Diplotaxis tenuifolia (L.) DC.	SB	SF
Echium vulgare L.	SB	BF
Erigeron karvinskianus DC.	SB	L
Erodium cicutarium (L.) L'Hér.	L	BF
Foeniculum vulgare Mill.	SB	SF
Fumaria capreolata L.	BF	L
Fumaria officinalis L.	HB	L
Galium verum L.	SB	BF
Geranium molle L.	SF	TF
Geranium robertianum L.	SB	SF
Glechoma hederacea L.	HB	L
Hedera helix L.	SB	SF
Hedysarum coronarium L.	BB	SB

*Helichrysum italicum (*Roth) G.Don	SB	L
Hyoseris radiata L.	HB	L
Hypericum perforatum L.	BB	HB
Hypochaeris radicata L.	SB	L
Inula viscosa (L.) Aiton	HB	L
Lamium amplexicaule L.	SB	BB
Lamium maculatum L.	HB	SB
Lamium purpureum L.	SB	BB
Lavatera punctata All.	SB	L
Lobularia maritima (L.) Desv.	HB	SB
Lotus corniculatus L.	SB	L
Lunaria annua L.	SB	SF
Malva neglecta Wallr.	HB	L
Malva sylvestris L.	SB	L
Muscari botrioides (L.) Mill.	L	BF
Myosotis ramosissima Rochel	SB	L
Ornithogalum umbellatum L.	SB	SF
Oxalis articulata Savigny	HB	SF
Oxalis corniculata L.	SF	TF
Papaver rhoeas L.	HB	SF
Phytolacca americana L.	SB	BF
Picris echioides L.	HB	L
Picris hieracioides L.,	HB	L
Portulaca oleracea L.	HB	BF
Potentilla reptans L.	HB	BF
Poterium sanguisorba L.	SF	TF
Prunella vulgaris L.	SB	BB
Reichardia picroides (L.) Roth	HB	SF
Reseda alba L.	SF	BB
Romulea bulbocodium L.	SF	BB
Rubus ulmifolius Schott	SB	L
Salpichroa origanifolia (Lam.) Baill.	SB	L
Salvia verbenaca L.	L	SB
Scabiosa columbaria L.	HB	L
Scilla bifolia L.	L	HB
Scolymus hispanicus L.	HB	SF
Sedum album L.	SB	BB
Sedum rupestre L.	HB	L
Sherardia arvensis L.	SB	L
Silene latifolia Poir.	SB	HB
Solanum dulcamara L.	HB	SB
Solanum nigrum L.	HB	SF
Sonchus asper (L.) Hill	SB	SF
Sonchus oleraceus L.	SB	SF
Sonchus tenerrimus L.	SB	SF
Stellaria media (L.) Vill.	BF	SF
Symphytum officinale L.	SB	HB
Taraxacum officinale Weber	HB	SF
Torilis arvensis (Huds.) Link	TF	SB
Tragopogon pratensis L.	SF	L
Trifolium campestre Schreb.	HB	SB

Trifolium pratense L.	HB	SB
Trifolium repens L.	BB	SB
Urospermum dalechampii (L.) Sch.	SF	L
Valerianella locusta L.	SB	L
Verbascum blattaria L.	BB	HB
Verbascum sinuatum L.	BB	HB
Verbena officinalis L.	L	SB
Veronica cymbalaria Board	BF	SF
Veronica persica Poir.	BF	SF
Vinca major L.	SB	HB

1 HB=honeybees, SB=solitary bees, BB= bumblebees, SF=Syrphid flies, BF=bombyliid flies, TF= Tachinid flies, L=lepidoptera.
